# Soil‐borne legacy facilitates the dissemination of antibiotic resistance genes in soil–plant continua

**DOI:** 10.1002/imt2.70094

**Published:** 2025-11-23

**Authors:** Zufei Xiao, Kai Ding, Xiaodong Guo, Yi Zhao, Xinyuan Li, Daoyuan Jiang, Dong Zhu, Qinglin Chen, Mui‐Choo Jong, David W. Graham, Gang Li, Yong‐Guan Zhu

**Affiliations:** ^1^ State Key Laboratory of Regional and Urban Ecology, Institute of Urban Environment Chinese Academy of Sciences Xiamen China; ^2^ Zhejiang Key Laboratory of Pollution Control for Port‐Petrochemical Industry CAS Haixi Industrial Technology Innovation Center in Beilun Ningbo China; ^3^ Key Laboratory of Groundwater Conservation of MWR China University of Geosciences (Beijing) Beijing China; ^4^ School of Water Resources and Environment China University of Geosciences (Beijing) Beijing China; ^5^ Institute of Environment and Ecology, Tsinghua Shenzhen International Graduate School Tsinghua University Shenzhen China; ^6^ Department of Biosciences Durham University Durham UK

## Abstract

Antimicrobial resistance (AMR) disseminates throughout the soil–plant continuum via complex microbial interactions. Plants shape root‐ and leaf‐associated microbiomes that sustain plant health; however, soil‐borne legacies—enriched with antibiotic‐producing microbes and resistance genes—govern AMR dynamics across agroecosystems. Using 16S rRNA gene sequencing, shotgun metagenomics, and high‐throughput quantitative PCR, we profiled antibiotic resistance genes (ARGs), mobile genetic elements, and virulence factor genes across bulk soil, rhizosphere, phyllosphere, and root endosphere within soil–tomato and soil–strawberry continua. Recurrent bacterial wilt amplified the resistome, particularly polypeptide resistance genes, thereby establishing the rhizosphere as a major hotspot of ARG accumulation. Multidrug‐resistant *Ralstonia solanacearum* (*R. solanacearum*) strains acted as major ARG reservoirs, harboring resistance determinants on both chromosomes and megaplasmids. Collectively, these findings demonstrate that pathogen‐driven restructuring of the plant microbiome accelerates ARG dissemination, establishing soil‐borne diseases as critical amplifiers of AMR across agricultural ecosystems.

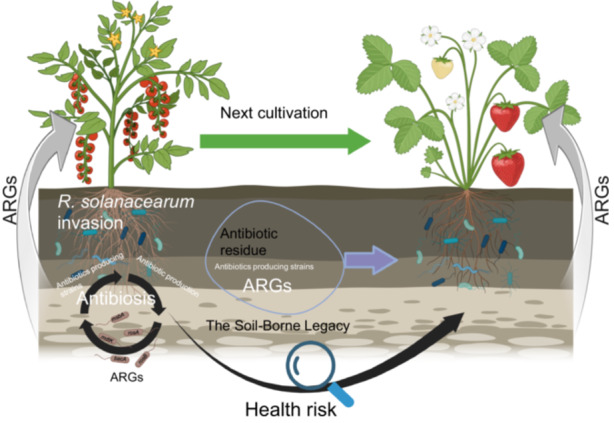

## ETHICS STATEMENT

No animals or humans were involved in this study.

To the Editor,

The spread of antibiotic resistance genes (ARGs) from soils to crops, livestock, and human environments poses a critical challenge to global health within the One Health framework [1, 2]. Research and policy focus on human‐centered systems such as hospitals, yet food systems—especially plant‐based production—remain underexplored despite their important role in antimicrobial resistance (AMR) emergence and dissemination [3]. Soils serve as reservoirs of ARGs and human pathogens [1]. Agricultural intensification, expansion of arable land, and the increasing prevalence of “superbugs” underscore the urgency of understanding AMR dynamics across soil–plant continua [4]. ARG risk assessment requires evaluating gene types, abundance, horizontal transfer potential (HGT), and host pathogenicity [5]. The co‐occurrence of ARGs with virulence factor genes (VFGs) and mobile genetic elements (MGEs) amplifies microbial risks.

Plants resist phytopathogens through a “cry‐for‐help” strategy that recruits root microbiota to form disease‐suppressive communities, also known as soil‐borne legacies. Disease‐suppressive soils allow plants to thrive despite phytopathogen presence [6]. Mechanisms of disease suppression include: (i) beneficial microbes occupying ecological niches and outcompeting pathogens for resources and space, thereby limiting their growth and spread; (ii) production of antimicrobial compounds (e.g., antibiotics) that inhibit proliferation, disrupt cellular integrity, and reduce virulence; and (iii) formation of symbiotic associations with plant roots, which, through signaling molecules or metabolites, induce systemic resistance. These processes activate host defenses, enabling rapid responses to pathogen invasion and limiting disease progression [7]. This study focuses on the ecological consequences of antimicrobial production (Mechanism ii). Antagonistic interactions among soil microbes frequently result from antibiotic production by specific strains in response to phytopathogens [8]. Consequently, an evolutionary “arms race” between plants and phytopathogens, together with antagonistic interactions between beneficial and pathogenic microbes, may accelerate AMR in plant‐associated microbiomes, including bulk soil, rhizosphere, root endosphere, and phyllosphere [9, 10]. The extensive use of antibiotics and biocontrol agents in agriculture has further elevated resistance levels in phytopathogens [11]. Such intrinsic resistance traits allow selective isolation of *Ralstonia solanacearum* (*R. solanacearum*) in antibiotic‐containing media. Moreover, its presence may indirectly enhance the AMR potential of neighboring microbial communities, including other pathogens that acquire ARGs [12]. However, the extent to which increasing resistance in *R. solanacearum* shapes the broader development of AMR across the soil–plant continuum remains unclear.

**Figure 1:**
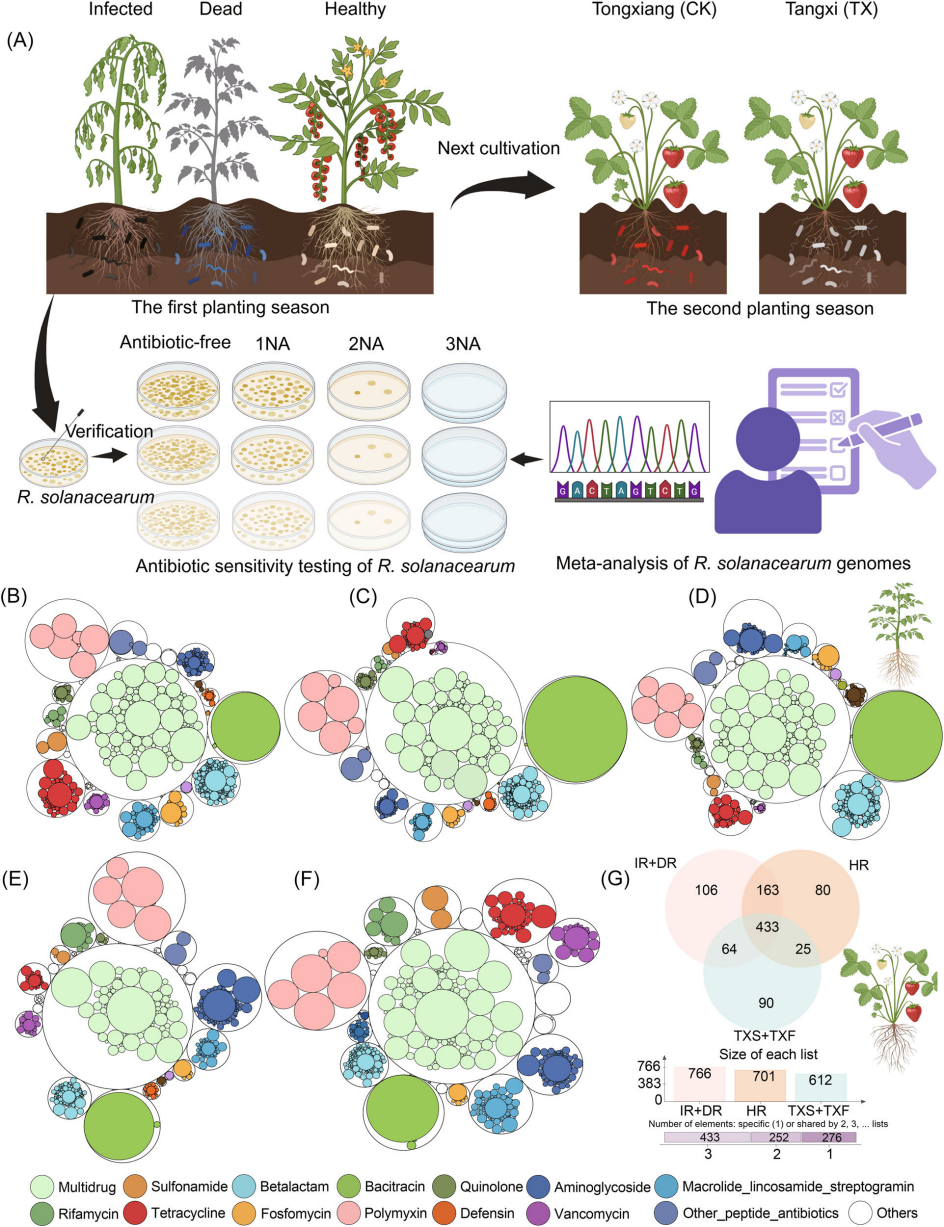
Soil‐borne legacies facilitate the dissemination of ARGs in soil–plant continua. (A) Experimental flowchart. The study consisted of four experiments: two field investigations and two confirmatory assays. In the first field study, the resistome and microbiome—including bacteria, ARGs, VFGs, MGEs, and metabolic pathways—were characterized in the soil–tomato continuum across diseased, healthy, and dying plants under field conditions. The second field study assessed the resistome in strawberry plants cultivated in the same soils following tomato harvest. In the first confirmatory assay, antibiotic resistance of *R. solanacearum* isolates was evaluated through sensitivity testing. In the second confirmatory assay, genomic analysis was conducted to identify ARGs in *R. solanacearum* isolates and to assess multidrug resistance. (B–D) Distribution and composition of the antibiotic resistome in the soil–tomato continuum across plant health states. Resistome composition is shown by type, with inner circles representing ARG subtypes and outer circles representing ARG types. Circle size indicates normalized ARG abundance in healthy (B), infected (C), and dead (D) groups. (E and F) Distribution, composition, and sources of ARGs in soil–strawberry continua across ecological niches. Resistome composition is illustrated with inner and outer circles representing ARG subtypes and types, respectively; circle size indicates normalized ARG abundance in CK (E) and TX (F) groups. (G) Source‐tracking analysis predicting the origins of ARGs in soil–strawberry continua across samples. NA, nutrient agar medium; HR, rhizosphere soil of healthy tomatoes; IR, rhizosphere soil of infected tomatoes; DR, rhizosphere soil of dead tomatoes; CK, soil–strawberry continuum from Tongxiang with no history of bacterial wilt; TX, soil–strawberry continuum from Tangxi with a history of bacterial wilt; TXF, strawberry fruits of TX; TXS, rhizosphere soil of TX.

This study investigates bacterial wilt caused by *R. solanacearum*, a pathogen inherently resistant to multiple antibiotics. We analyzed a tomato greenhouse with a long history of bacterial wilt outbreaks, which later spread to strawberry crops, indicating a persistent soil‐borne legacy. We hypothesized that bacterial wilt caused by *R. solanacearum* imposes selective pressures that progressively enrich specific ARGs, with effects extending beyond the soil–tomato continuum to subsequent systems such as the soil–strawberry continuum. The objectives of this study were to: (i) monitor changes in the resistome and microbiome during *R. solanacearum* infection, (ii) determine whether ARGs are transferred to a subsequent soil–strawberry continuum, and (iii) assess exposure risks by identifying pathogens carrying both ARGs and MGEs (Figure 1A). Using high‐throughput quantitative PCR (HT‐qPCR) and shotgun metagenomics, we profiled ARGs, VFGs, and MGEs across soil–plant interfaces to clarify the ecological impact of bacterial wilt on AMR dissemination in agricultural systems.

## SHIFTS IN THE BACTERIAL COMMUNITY UNDER *R. SOLANACEARUM* INVASION

Microbial community analyses showed that invasion by *R. solanacearum* markedly reduced bacterial richness and diversity, causing pronounced shifts in rhizosphere, phyllosphere, and root endosphere communities (Figure S1). Dominant taxa—including Gammaproteobacteria, Alphaproteobacteria, Bacilli, and Actinobacteria—exhibited infection‐dependent shifts in relative abundance (Figures S1 and S2). These findings highlight the disruptive effect of bacterial wilt on the stability of plant‐associated microbial communities. Reduced diversity diminishes ecological buffering capacity, enabling pathogen‐driven selection to reshape community composition. Even in asymptomatic plants, phytopathogen presence triggers subtle antagonistic interactions, recruiting beneficial microbes that suppress disease. This microbial “arms race” inadvertently enriches antibiotic‐producing microbes and, consequently, ARGs (Figures S3 and S4). Thus, pathogen‐driven microbiome restructuring emerges as a key ecological mechanism driving resistome amplification. Previous studies have shown that healthy plant microbiomes harbor abundant antibiotic biosynthesis genes, such as polyketide synthases and nonribosomal peptide synthetases, suggesting that microbial antibiosis plays a central role in shaping soil‐borne legacies [13].

## RESISTOME RESHAPING ACROSS THE SOIL–TOMATO CONTINUUM

Metagenomic profiling identified 1334 ARG subtypes across 29 categories, with richness highest in healthy plants, followed by infected and dead plants (Tables S1 and S2). The resistome was primarily dominated by multidrug, bacitracin, polymyxin, and β‐lactam resistance genes (Figure 1B–D). In all conditions, the rhizosphere consistently exhibited the highest abundance and diversity of ARGs, MGEs, and VFGs, confirming its role as a hotspot for resistance dissemination (Figures S5–S7). These results suggest that although beneficial microbes suppress disease and support plant health, they simultaneously impose strong antibiotic selection, enriching ARG reservoirs (Figures S8–S10). By contrast, phytopathogen infection promoted MGE mobilization and enrichment of clinically relevant ARGs, indicating that pathogen‐induced stress enhances HGT (Figure S11).

Within the soil–plant continuum, ARGs spread through three interconnected pathways: (i) atmospheric deposition and uptake via stomata or root wounds; (ii) dominance of antibiotic‐resistant bacteria in the rhizosphere, followed by HGT to endophytes migrating through the vascular system; and (iii) transfer via soil fauna such as earthworms, which recycle ARGs through ingestion and excretion [14]. These processes establish bidirectional gene flow between soil bacteria and endophytes, enabling ARGs to enter the food chain and ultimately pose risks to human health. Collectively, these findings support the view that both resistant and diseased systems expand the resistome, albeit through distinct ecological mechanisms.

Antibiotic susceptibility testing further revealed variable resistance to polymyxin B, bacitracin, chloramphenicol, penicillin, tetracycline, and actinomycin D (Figure S12). Isolation of highly pathogenic, multidrug‐resistant *R. solanacearum* strains confirmed its role as a major reservoir of ARGs. Genomic meta‐analysis revealed that both chromosomal and plasmid elements of *R. solanacearum* encode resistance to a wide range of antibiotic classes, including polymyxins, bacitracin, macrolides, tetracyclines, fluoroquinolones, aminocoumarins, monobactams, elfamycins, and aminoglycosides (Table S3). Combined genotypic and phenotypic resistance analyses highlight a direct pathway by which pathogen‐driven selection amplifies clinically relevant traits in the soil–plant continuum, posing concrete food safety risks. Multi‐model integration indicates that *R. solanacearum* drives ARG enrichment through both direct and indirect ecological mechanisms, with MGEs acting as key mediators of mobilization (Figure S13). This study highlights the interconnected roles of pathogen pressure, microbial interactions, and HGT in shaping resistome dynamics in agricultural ecosystems.

## SOIL‐BORNE LEGACY AND CROSS‐CROP ARG DISSEMINATION

Analysis of the soil–strawberry continuum demonstrated that bacterial wilt legacies extended beyond the tomato system (Figure 1E,F). Strawberry plants cultivated in previously infected soils exhibited significantly higher ARG abundance and diversity in fruits compared to controls (Figures S14A and S15, Table S4). Both HT‐qPCR and metagenomic sequencing confirmed that soil‐borne legacies of bacterial wilt increase the potential for ARG translocation into plant tissues (Figure S16). Source tracking revealed that 66%–89% of ARGs in strawberry fruits originated from healthy tomato rhizospheres, indicating direct cross‐species transfer (Figure 1G and Figure S14B). This evidence confirms that soil‐borne legacies act as reservoirs that facilitate ARG migration between crops, thereby expanding the ecological footprint of bacterial wilt. Notably, even asymptomatic tomato rhizospheres acted as primary ARG donors, indicating that resistance risks persist despite the absence of visible disease. These findings have direct implications for food safety, as edible tissues can accumulate ARGs derived from past phytopathogen pressures.

Although plant health reflects suppression of *R. solanacearum*, it does not eliminate continued pathogen‐driven selection. Instead, the asymptomatic phase is characterized by intense, subclinical microbial warfare [13]. During a “cry for help” response, plants recruit beneficial microbes that produce antimicrobial compounds to suppress phytopathogens [15]. However, this process exerts strong selective pressure, enriching resistance traits in both phytopathogens and beneficial microbes. Under these conditions, HGT among phytopathogens, symbionts, and commensals is likely accelerated, promoting ARG accumulation on MGEs in the rhizosphere. Death of phytopathogens further facilitates ARG dissemination across the soil–plant continuum by releasing cellular contents, reshaping microbial communities, accelerating tissue decomposition, and enabling HGT [11, 16, 17].

The soil‐borne legacy, also termed inhibitory soil memory, supports healthy plant growth while simultaneously driving ARG dissemination in the soil–strawberry continuum, likely due to the high abundance of antimicrobial‐producing bacteria and multiple ARGs [18]. This effect is attributed to the enrichment of antimicrobial‐producing bacteria, including *Actinobacteria*, *Bacillus*, *Pseudomonas*, and *Lysobacter*, which harbor biosynthetic gene clusters and ARGs. Together with *Streptomyces*, these microbes promote antibiotic biosynthesis and disease suppression, while also acting as ARG reservoirs [5, 13]. Positive correlations among ARGs, MGEs, and *R. solanacearum* indicate that bacterial wilt serves as a vehicle for ARG dissemination across the soil–plant continuum, posing serious risks to food safety and human health and highlighting the need for enhanced monitoring (Figures S11 and S17).

## MOBILIZATION OF ARGS AND PUBLIC HEALTH RISKS

**Figure 2:**
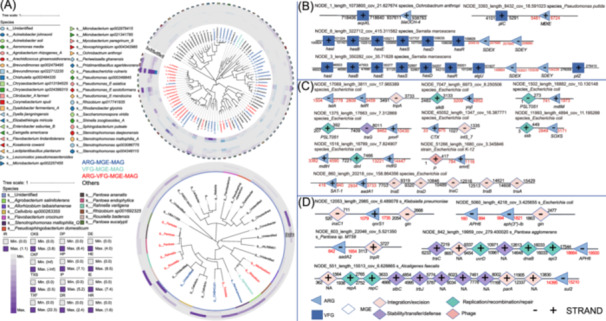
Microbial hosts carrying ARGs, MGEs, and VFGs in soil–tomato and soil–strawberry continua. (A) Phylogenetic trees of MAGs from the soil–tomato (Upper left) and soil–strawberry (Lower left) continua, colored by ARG‐MGE, VFG‐MGE, ARG‐VFG‐MGE carriers, and others. (B) Representative contigs carrying ARG‐MGE clusters and their putative hosts in the soil–tomato continuum (NCBI best hits). (C) Representative contigs carrying ARG‐VFG clusters and their putative host (*Escherichia coli*) in the soil–tomato continuum. (D) Representative contigs carrying ARG‐MGE clusters and their putative hosts in the soil–strawberry continuum. The numerical values denote the starting positions of the genes. The red and black coloring corresponds to ARGs and other genes, respectively. Abbreviations: Tomato continuum – HR, rhizosphere of healthy plants; HE, root endosphere of healthy plants; HP, phyllosphere of healthy plants; IR, rhizosphere of infected plants; IE, root endosphere of infected plants; IP, phyllosphere of infected plants; DR, rhizosphere of dead plants; DE, root endosphere of dead plants; DP, phyllosphere of dead plants; InR, rhizosphere before disease onset. Strawberry continuum – CKF, fruits from disease‐free Tongxiang; CKS, rhizosphere soil from disease‐free Tongxiang; TXF, fruits from Tangxi; TXS, rhizosphere soil from Tangxi with bacterial wilt history.

Metagenome‐assembled genome (MAG) analyses revealed that ARGs frequently co‐localize with MGEs and VFGs in both pathogens (e.g., *Escherichia coli*, *Klebsiella pneumoniae*) and beneficial taxa (e.g., *Bacillus*, *Pseudomonas*) (Figure 2A, Tables S5–S10). Their co‐occurrence on the same contigs indicates strong mobilization potential, facilitating HGT across microbial guilds. At the contig level, we identified multidrug, β‐lactam, and aminoglycoside resistance genes physically linked to transposases, integrases, and plasmid replication genes, providing direct evidence of mobilization potential (Figure 2B–D, Tables S11–S14). Such co‐localization, especially in pathogens and commensals, suggests that agricultural soils—particularly those affected by bacterial wilt—act as reservoirs and conduits for HGT to human pathogens, thereby promoting the emergence of clinically resistant strains.

These findings highlight the “double‐edged sword” role of soil microbiomes: biocontrol organisms used for disease suppression may also act as ARG reservoirs, while pathogens acquire resistance traits that increase clinical significance. A critical concern is the enrichment of polypeptide resistance genes, which compromise last‐resort antibiotics such as polymyxins and vancomycin. Nonribosomal antimicrobial peptides (AMPs), previously considered low‐risk due to minimal toxicity and specific modes of action, are now compromised by the spread of resistance. Many pathogens and soil probiotics naturally produce AMPs to outcompete competitors, but resistance reduces their ecological and clinical efficacy [19]. Alarmingly, vancomycin resistance genes were also detected transferring from bacterial wilt‐affected soils to strawberry fruits, highlighting agricultural soils as critical ecological–clinical interfaces with direct implications for food safety and public health [20].

## INTEGRATIVE PERSPECTIVE

Collectively, our findings demonstrate that *R. solanacearum* facilitates ARG persistence and dissemination across the soil–tomato continuum, even during asymptomatic phases. Soil‐borne legacies of bacterial wilt extend these effects across crop cycles, facilitating ARG migration into subsequent crops and edible tissues. The co‐occurrence of ARGs, MGEs, and VFGs in diverse microbial hosts highlights multiple transmission pathways and emphasizes the urgent need to integrate plant pathology, microbial ecology, and One Health frameworks in AMR management. This study highlights how pathogen pressure, microbial interactions, and HGT collectively shape resistome dynamics in agricultural ecosystems. Furthermore, future development of microbial fertilizers and biocontrol agents should incorporate dual criteria, simultaneously evaluating pathogen suppression efficacy and minimal ARG burden, to mitigate their unintended contribution to the public health threat of antibiotic resistance.

However, several limitations should be considered. Reliance on short‐read metagenomic sequencing limits the resolution of MGEs, such as plasmids, restricting accurate assignment of ARGs and MGEs to their hosts. Environmental factors—including soil pH, organic matter, and pesticide use—were not independently controlled and may have influenced ARG distributions despite standardized management practices. As a cross‐sectional study, we could not capture long‐term ARG persistence or microbial community dynamics. Additionally, both metagenomics and HT‐qPCR are limited by host DNA contamination, complicating absolute quantification. While metagenomics provides broad ARG profiles, HT‐qPCR offers higher sensitivity for rare, high‐risk ARGs, and methodological differences likely explain minor inconsistencies.

Future research should prioritize: (i) applying long‐read sequencing or Hi‐C–based binning to resolve ARG–MGE–host genomic contexts, (ii) integrating metatranscriptomics and functional assays to examine active ARG expression and microbial interactions under pathogen stress, (iii) conducting long‐term monitoring and time‐series analyses to track ARG dynamics across seasons and management practices, and (iv) implementing controlled experiments to disentangle the effects of environmental factors such as organic matter and pesticide residues on resistome dissemination.

## AUTHOR CONTRIBUTIONS

**Zufei Xiao**: Conceptualization; methodology; software; data curation; investigation; validation; formal analysis; visualization; writing—original draft; writing—review and editing. **Kai Ding**: Conceptualization; methodology; validation; investigation; formal analysis; funding acquisition; project administration; resources; writing—original draft; writing—review and editing. **Xiaodong Guo**: Investigation; validation; writing—original draft; writing—review and editing. **Yi Zhao**: Writing—original draft; writing—review and editing; methodology. **Xinyuan Li**: Writing—original draft; writing—review and editing; methodology. **Daoyuan Jiang**: Writing—original draft; writing—review and editing; investigation. **Dong Zhu**: Writing—original draft; writing—review and editing; validation; methodology. **Qinglin Chen**: Writing—original draft; writing—review and editing; visualization. **Mui‐Choo Jong**: Methodology; validation; writing—original draft; writing—review and editing; data curation; supervision; software; formal analysis. **David W. Graham**: Writing—original draft; writing—review and editing; methodology; conceptualization. **Gang Li**: Conceptualization; methodology; writing—original draft; writing—review and editing; validation; investigation; supervision; project administration; resources; funding acquisition. **Yong‐Guan Zhu**: Conceptualization; methodology; supervision; formal analysis; validation; writing—original draft; writing—review and editing; resources. All authors have read the final manuscript and approved it for publication.

## CONFLICT OF INTEREST STATEMENT

The authors declare no conflicts of interest.

## Supporting information


**Figure S1.** Response of bacterial communities to R. solanacearum infestation in the soil–tomato continuum.
**Figure S2.** Bacterial community responses to R. solanacearum infestation in the soil–tomato continuum.
**Figure S3.** Relative abundance of Bacillus subtilis and Bacillus velezensis in rhizosphere soils from healthy, infected, and dead tomato plants.
**Figure S4.** Differential metabolic pathways between healthy and infected plants in the soil–tomato continuum.
**Figure S5.** Relative abundance of ARG types across plant health states based on metagenomic analysis.
**Figure S6.** Distribution and composition of MGEs in the soil–tomato and soil–strawberry continua.
**Figure S7.** Distribution and composition of VFGs in the soil–tomato and soil–strawberry continua.
**Figure S8.** Differential ARG types between healthy and infected plants in the soil–tomato continuum.
**Figure S9.** Response of MGEs and VFGs to R. solanacearum infestation.
**Figure S10.** Differential ARG profiles between healthy and infected plants based on HT‐qPCR.
**Figure S11.** Linear regression analysis linking ARGs with MGEs and R. solanacearum.
**Figure S12.** Antibiotic susceptibility test results for R. solanacearum.
**Figure S13.** Potential drivers of ARG variation in the soil–plant continuum identified by metagenomic and HT‐qPCR analyses.
**Figure S14.** Distribution and niche‐specific patterns of ARGs revealed by metagenomic analysis.
**Figure S15.** Heatmap illustrating the top 50 most abundant ARG subtypes in the soil–strawberry continuum.
**Figure S16.** Distribution and composition of ARGs in the soil–strawberry continuum.
**Figure S17.** Linear regression analysis of the relationship between ARG relative abundance and ARG‐host relative abundance in the soil–tomato (A) and soil–strawberry (B) continua.
**Figure S18.** Absolute abundance of R. solanacearum in rhizosphere soil (A) and root endophytes (B) of healthy, infected, and dead tomato plants.
**Figure S19.** Schematic of fractionation protocol.

**Table S1.** Summary table of key ARGs by niche.
**Table S2.** Summary of ARGs identified by ARG‐OAP analysis based on metagenomic sequencing across the soil–tomato continuum.
**Table S3.** Meta‐analysis result of R. solanacearum genomes.
**Table S4.** Summary of MGEs identified by ARG‐OAP analysis based on metagenomic sequencing across the soil–strawberry continuum.
**Table S5.** Distribution of ARGs identified within MAGs from the soil–tomato continuum.
**Table S6.** Distribution of VFGs identified within MAGs from the soil–tomato continuum.
**Table S7.** Distribution of MGEs identified within MAGs from the soil–tomato continuum.
**Table S8.** Distribution of ARGs identified within MAGs from the soil–strawberry continuum.
**Table S9.** Distribution of VFGs identified within MAGs from the soil–strawberry continuum.
**Table S10.** Distribution of MGEs identified within MAGs from the soil–strawberry continuum.
**Table S11.** Contigs carrying ARGs identified from metagenomic assemblies of the soil–tomato continuum.
**Table S12.** Contigs carrying VFGs identified from metagenomic assemblies of the soil–tomato continuum.
**Table S13.** Contigs carrying ARGs identified from metagenomic assemblies of the soil‐strawberry continuum.
**Table S14.** Contigs carrying VFGs identified from metagenomic assemblies of the soil‐strawberry continuum.
**Table S15.** The specific naming strategy for the samples in this study.
**Table S16.** Primers used in this study.
**Table S17.** PCR amplification program and reaction system for this study.
**Table S18.** Raw reads and cleaned reads for shotgun metagenomic sequencing.
**Table S19.** Detailed information on R. solanacearum strains used for genomic meta‐analysis.
**Table S20.** List of abbreviations.

## Data Availability

The raw amplicon and metagenomic data were archived in the Genome Sequence Archive at the BIG Data Center, Chinese Academy of Sciences, and assigned accession numbers CRA020401 and CRA020305, https://ngdc.cncb.ac.cn/gsa/browse/CRA020305 and https://ngdc.cncb.ac.cn/gsa/browse/CRA020401. All code and algorithms used in this study are published and cited in the Methods section. The data and scripts used are saved in GitHub https://github.com/XiaoRhywings/Soil-borne-legacy-spreads-antibiotic-resistance. Supplementary materials (methods, figures, tables, graphical abstract, slides, videos, Chinese translated version, and update materials) may be found in the online DOI or iMeta Science http://www.imeta.science/. The data that support the findings of this study are openly available in the Genome Sequence Archive at the BIG Data Center, Chinese at https://ngdc.cncb.ac.cn/gsa, reference numbers CRA020401 and CRA020305.
