# Supplementary material for: Soil‐borne legacy facilitates the dissemination of antibiotic resistance genes in soil–plant continua
Source: Imeta. 2025 Nov 23;4(6):e70094. doi: 10.1002/imt2.70094 (PMC12747550; doi:10.1002/imt2.70094)
Supplement: Supplementary file 1 — Figure S1. Response of bacterial communities to R. solanacearum infestation in the soil–tomato continuum. Figure S2. Bacterial community responses to R. solanacearum infestation in the soil–tomato continuum. Figure S3. Relative abundance of Bacillus subtilis and Bacillus velezensis in rhizosphere soils from healthy, infected, and dead tomato plants. Figure S4. Differential metabolic pathways between healthy and infected plants in the soil–tomato continuum. Figure S5. Relative abundance of ARG types across plant health states based on metagenomic analysis. Figure S6. Distribution and composition of MGEs in the soil–tomato and soil–strawberry continua. Figure S7. Distribution and composition of VFGs in the soil–tomato and soil–strawberry continua. Figure S8. Differential ARG types between healthy and infected plants in the soil–tomato continuum. Figure S9. Response of MGEs and VFGs to R. solanacearum infestation. Figure S10. Differential ARG profiles between healthy and infected plants based on HT‐qPCR. Figure S11. Linear regression analysis linking ARGs with MGEs and R. solanacearum. Figure S12. Antibiotic susceptibility test results for R. solanacearum. Figure S13. Potential drivers of ARG variation in the soil–plant continuum identified by metagenomic and HT‐qPCR analyses. Figure S14. Distribution and niche‐specific patterns of ARGs revealed by metagenomic analysis. Figure S15. Heatmap illustrating the top 50 most abundant ARG subtypes in the soil–strawberry continuum. Figure S16. Distribution and composition of ARGs in the soil–strawberry continuum. Figure S17. Linear regression analysis of the relationship between ARG relative abundance and ARG‐host relative abundance in the soil–tomato (A) and soil–strawberry (B) continua. Figure S18. Absolute abundance of R. solanacearum in rhizosphere soil (A) and root endophytes (B) of healthy, infected, and dead tomato plants. Figure S19. Schematic of fractionation protocol. [file IMT2-4-e70094-s002.docx]

Supporting information to

**Soil-borne legacy facilitates the dissemination of antibiotic resistance genes in soil–plant** **continua**

**Running title:** Soil-borne legacy spreads antibiotic resistance

Zufei Xiao^1, 2#^, Kai Ding^1, 2#^, Xiaodong Guo^1, 2^, Yi Zhao^3, 4^, Xinyuan Li^1, 2^, Daoyuan Jiang^1, 2^, Dong Zhu^1, 2^, Qinglin Chen^1, 2^, Mui-Choo Jong^5*^, David W Graham^1, 6^, Gang Li^1, 2*^, Yong-Guan Zhu^1, 2^

^1^State Key Laboratory of Regional and Urban Ecology, Institute of Urban Environment, Chinese Academy of Sciences, Xiamen 361021, China

^2^Zhejiang Key Laboratory of Pollution Control for Port-Petrochemical Industry, CAS Haixi Industrial Technology Innovation Center in Beilun, Ningbo 315830, China

^3^Key Laboratory of Groundwater Conservation of MWR, China University of Geosciences (Beijing), Beijing 100083, China

^4^School of Water Resources and Environment, China University of Geosciences (Beijing), Beijing 100083, China

^5^Institute of Environment and Ecology, Tsinghua Shenzhen International Graduate School, Tsinghua University, Shenzhen 518055, China

^6^Department of Biosciences, Durham University, Durham DH1 3LE, United Kingdom

^#^These authors contributed equally: Zufei Xiao, Kai Ding.

*Correspondence: gli@iue.ac.cn (Gang Li); [florence.jong@sz.tsinghua.edu.cn](mailto:florence.jong@sz.tsinghua.edu.cn) (Mui-Choo Jong)

**METHODS**

**Field site and sampling strategy**

The field investigation was carried out in a suburban agricultural area in Tangxi, Ningbo, Zhejiang Province, China (121°42′15″ E, 29°40′48″ N). The site had been continuously used for high-intensity cultivation for more than a decade, following a rotation system of Solanaceae crops (October–June), lettuce or Chinese cabbage (July–September), and strawberry (October–May). Sampling was performed in May 2022 (premorbid soil–tomato continuum), June 2022 (diseased soil–tomato continuum), and March 2023 (soil–strawberry continuum). A disease-free strawberry plantation in Tongxiang, Jiaxing, Zhejiang Province, China (120°33′8″ E, 30°29′46.96″ N), was selected as the control site due to its comparable soil properties, fertilization practices, and cultivation history. Soil–tomato continuum samples were fractionated into bulk soil, rhizosphere soil, phyllosphere, and root endosphere, while soil–strawberry continuum samples comprised rhizosphere soil and fruits (Table S15).

**Sampling and pretreatment strategy**

Sampling was performed at 2:00 PM under environmental conditions of 28–29 °C, 5.1–6.0 mm rainfall, and 90%–95% relative humidity. Plants were classified into three pathological states: (i) dead due to *R. solanacearum* infection; (ii) infected with wilted or weakened leaves and basal stem discoloration; and (iii) healthy, with turgid leaves and no visible symptoms on stems or leaves. Latent infections were excluded by performing qPCR on rhizosphere soil and root endophytes (Figure S18). To minimize bias, a standardized protocol was applied: (i) all samples were collected at the same soil depth and growth stage; (ii) soil type, crop variety, and field management were consistent across sites within each treatment group; and (iii) composite soil sampling (pooling subsamples from multiple points within a plot) was used to capture spatial heterogeneity.

Tomato roots were gently shaken to remove loosely adhering soil, which was designated as bulk soil. Soil tightly adhering to the root surface was defined as rhizosphere soil. Each sample type, representing distinct soil–plant compartments, was collected in six biological replicates. Rhizosphere and phyllosphere samples were processed within 8 h of collection (Figure S19). Bulk soil samples were split into two subsamples: one stored at −20 °C for physicochemical analysis and the other at −80 °C for microbial analysis.

**DNA extraction, 16S rRNA gene sequencing, and high-throughput qPCR**

Total DNA was extracted from all samples using a FastDNA^®^ SPIN Kit for Soil (MP Biomedicals, Solon, USA). DNA concentration and purity assessed with a Qubit^™^ 3.0 fluorometer and Nanodrop 2000 spectrophotometer (Thermo Fisher Scientific Inc., Waltham, USA), diluted to 20 ng/μl, and stored at -80 °C

Pathogen abundance was quantified by qPCR targeting the *fli*C of *R. solanacearum* [1]. The V3–V4 region of the 16S rRNA gene was amplified with the 338F/806R primer pair (Table S16). Detailed sequencing and analysis protocols followed published methods (Table S17) [2].

High-throughput qPCR was conducted with 384 primer pairs targeting 319 ARGs, the 16S rRNA gene, and 59 MGEs, including 24 MGEs, 9 insertion sequences, 10 transposase genes, 11 plasmids, and 3 integrase genes [3]. qPCR data were processed in SmartChip qPCR software v2.7.0.1. Wells showing multiple melting peaks or amplification efficiencies outside 1.8–2.2 were excluded. A threshold cycle (Ct) of 31 was set as the detection limit. Only samples with successful amplification in all three technical replicates were considered positive for downstream analysis. Relative copy numbers were calculated as: relative gene copy number = 10^(31−Ct) (10/3)^, where Ct equals the threshold cycle. ARG relative abundance was calculated to minimize biases from host DNA contamination and microbial load variability [4].

**The formula for calculating the relative abundance of ARGs**

*R_i_* _ARG-like reads_ represent the total aligned length of all reads assigned to the *i*-th ARG reference sequence; *L_i_* _ARG referen ce sequence_ refers to the nucleotide sequence length of the *i*-th reference sequence in the ARG database; *R_jk_* _marker-like reads_ refer to the total aligned length of all reads mapped to the *j*-th reference sequence in the *k*-th marker gene of essential bacterial (or prokaryotic) single-copy marker genes (ESCMGs), typically including 30 genes; *L_jk_* _marker gene_ refers to the nucleotide sequence length of the *j*-th reference sequence in the *k*-th marker gene cluster of ESCMGs or single copy genes (SCGs).

$$\mathrm{Abundance}=\frac{\sum_{i=1}^{n} R_{i ARG-like reads}/L_{i ARG reference sequence}}{\frac{1}{30}\times\sum_{k=1}^{30} \sum_{j=1}^{m} R_{jk marker-like reads}/L_{jk maker gene}}$$

**Shotgun metagenomics sequencing and functional analysis**

Based on 16S profiles, shotgun metagenomic sequencing was performed on 52 samples spanning rhizosphere soil, phyllosphere, and root endosphere (soil–tomato continuum), as well as rhizosphere soil and fruits (soil–strawberry continuum). Sequencing (≈ 15 Gb per sample, 2 × 150 bp paired-end) was conducted by MAGIGENE (Guangzhou, China) (Table S18).

Metagenomic datasets were processed using our in-house bioinformatics pipeline, EasyMetagenome—an intuitive and flexible platform tailored for shotgun metagenomic analysis in microbiome research (<https://github.com/XiaoRhywings/EasyMetagenome>) [5]. Raw read quality was assessed with FastQC (v0.12.1), and results were summarized using MultiQC (v1.15) [6]. Raw reads were preprocessed with fastp (v0.21.0), including quality trimming and host DNA removal using KneadData (v0.12.0). Cleaned reads were reassessed for quality using FastQC (v0.12.1) and MultiQC (v1.15). Taxonomic and metabolic pathway profiles were inferred for each sample using MetaPhlAn (v4.0.6) [7] and HUMAnN (v3.7) [8]. Feature counts were converted to transcripts per million (TPM) and divided by the gigabase of quality-filtered reads (TPM/Gbp). Cleaned paired-end reads were analyzed for ARGs using ARGs-OAP (v3.2.4, default parameters) with the structured ARG (SARG, v3.2.1) database [9]. “ARG copies per cell” was used as the standard unit in all subsequent analyses [10].

MetaSPAdes (v3.15.4) was used to assemble short reads into longer contigs for each sample [11]. All assemblies were subsequently merged. Gene prediction was carried out with the meta-mode of Prodigal (v2.6.3), followed by clustering and redundancy removal with CD-HIT (v4.8.1). Binning was conducted with MetaWRAP (v1.3.2) using three algorithms: MetaBAT (v2.12.1), MaxBin (v2.2.6), and CONCOCT (v1.0.0) [12]. Bins were assessed with CheckM2 (v1.0.1) and retained for pairwise dereplication if classified as high quality (≥ 90% completeness, ≤ 5% contamination) or medium quality (≥ 70% completeness, ≤ 10% contamination) [13]. Taxonomic classification of each metagenome-assembled genome (MAG) was assigned using GTDB-Tk (v2.3, classify_wf function). Open reading frames (ORFs) were predicted from MAGs with Prodigal (v2.6.3). Phylogenetic relationships among MAGs were inferred with FastTree (v2.1.11) using 120 bacterial domain-specific marker genes from GTDB. Contigs and MAGs were annotated for ARGs, VFGs, MGEs, and taxonomic assignment using RGI (v6.0.3) [14], DeepARG (v1.0.2) [15], ABRicate (v1.0.1, https://github.com/tseemann/abricate), mobileOG-db (beatrix-1.6) [16], and MMseqs2 (v15.6f452) [17]. Matches were considered valid at ≥ 80% sequence identity and ≥ 70% sequence coverage.

**Meta-analysis of *R. solanacearum* genomes and antibiotic susceptibility assays**

Genomic datasets of *R. solanacearum* were compiled via (i) Google Scholar searches (“*Ralstonia solanacearum*,” “*Pseudomonas solanacearum*”) to retrieve accession numbers from complete genome studies, and (ii) queries to NCBI, NGDC, DDBJ, and ENA, yielding 1118 complete genomes (Table S19). ARGs were annotated with RGI and ABRicate, and results consolidated across datasets.

Antibiotic susceptibility assays were conducted on three virulent *R. solanacearum* strains isolated in this study. Nutrient agar (NA) was supplemented per 100 ml with: 10 mg polymyxin B sulfate, 2.5 mg bacitracin, 0.5 mg chloramphenicol, 0.5 mg penicillin, 5 mg tetracycline, 10 mg actinomycin, and 5 mg tetrazolium red, designated as 1NA. Media with 2- and 3-fold concentrations (designated 2NA and 3NA) were also prepared to assess sensitivity under elevated antibiotic pressure.

**Statistical analysis**

All statistical analyses were performed in R v4.2.3. Before α-diversity calculation, samples were rarefied to 9000 reads (10,000 iterations) using the ‘rrarefy’ function in vegan v2.6-4. Taxonomic profiles of ARGs, MGEs, and virulence factors were visualized using Maptree, integrating *RColorBrewer*, *ggtree*, *phyloseq*, *data.tree*, *viridis*, *tidyverse*, *ggraph*, and *igraph* [18]. Phylogenetic trees were constructed using *iTOL* [19]. Structural equation modeling (SEM) was performed using IBM SPSS Amos 26.0 with the maximum likelihood estimation method to test hypothesized pathways among latent variables. Model goodness-of-fit was assessed using multiple indices, including χ², df, CFI, TLI, RMSEA, and SRMR [20]. Results were visualized using *ggplot2*.

Boxplots were generated using *ImageGP* [21]. All data points were retained in the statistical analyses, with no exclusion of potential outliers. Box plots were used to visualize data distributions, defining outliers as values below Q1 − 1.5×IQR or above Q3 + 1.5×IQR. To ensure robust statistical inference, normality and homogeneity of variances were tested using the Shapiro–Wilk and Bartlett tests, respectively. Depending on the data distribution, either ANOVA followed by Tukey’s HSD post-hoc test or the Kruskal–Wallis test with Dunn’s multiple comparisons was applied. All pairwise comparisons included appropriate multiple testing corrections, ensuring the integrity of the complete dataset throughout the analyses.

Community composition was compared across niches and plant health conditions using Adonis2, PCoA, and CPCoA (*vegan* and *ggplot2*). Community dissimilarity was tested with Adonis2 (*vegan*, Bray–Curtis distance, 9999 permutations, strata = sampling_batch). Homogeneity of dispersion was verified before interpreting PERMANOVA results. Gene-gene associations were evaluated by linear regression and correlation analyses. Because ARG relative abundances are compositional, differential testing was performed with ALDEx2 v1.26.0 (clr transformation, 128 Dirichlet Monte-Carlo instances). Only features with *q* < 0.05 (Benjamini–Hochberg) and |effect-size| > 1 were considered significant.

Heatmaps were generated with the R package *pheatmap*, and volcano plots of DEGs were visualized using *EnhancedVolcano*. The Benjamini–Hochberg (BH) method was first applied to control the false discovery rate (FDR) in ARG analyses, with *q* < 0.05 considered significant. No ARGs remained significant after FDR correction. This pattern may be attributed to ARG abundance being normalized as copies per cell. To capture potential biological signals, we implemented a hierarchical confidence framework. Results with uncorrected *p* < 0.05 were classified as high-confidence candidates (meeting all three criteria) or medium-confidence candidates (meeting two). Classification was based on effect size (fold change > 2 or < 0.5) and biological plausibility (literature support or consistency with known resistance mechanisms). Only these two categories were discussed further, with their confidence level, raw p-value, q-value, and effect size explicitly reported. This approach enables cautious identification of ARGs with potential biological relevance while maintaining statistical rigor.

Beta-diversity was quantified using two axes from non-metric multidimensional scaling (NMDS) of Bray–Curtis dissimilarities in the ZOTU (Zero-radius Operational Taxonomic Unit) and ARG matrices. Key predictors influencing ARGs were identified using a random forest (RF) classification analysis. Variable importance was determined by the percentage increase in mean squared error (MSE), where higher MSE% indicates greater predictor importance. Model significance and cross-validated R² were evaluated with 5000 permutations of the response variable using the *A3* package. Predictor significance was further tested using the *rfPermute* package. Multiple regression with variance decomposition validated the RF results using the *lm* and *calc.relimp* functions in the *relaimpo* package [22]. Table S20 lists the full names of all abbreviations used in this study.

**REFERENCES**

1. Wei, Zhong, Yian Gu, Ville-Petri Friman, George A. Kowalchuk, Yangchun Xu, Qirong Shen, Alexandre Jousset. 2019. “Initial soil microbiome composition and functioning predetermine future plant health.” *Science Advances* 5: eaaw0759. [https://doi.org/10.1126/sciadv.aaw0759](https://doi.org/https://doi.org/10.1126/sciadv.aaw0759)

2. Xiao, Zufei, Changyi Lu, Zhiyong Wu, Xinyuan Li, Kai Ding, Zhe Zhu, Ruixia Han, et al. 2024. “Continuous cropping disorders of eggplants (*Solanum melongena* L.) and tomatoes (*Solanum lycopersicum* L.) in suburban agriculture: Microbial structure and assembly processes.” *Science of the Total Environment* 909: 168558. [https://doi.org/10.1016/j.scitotenv.2023.168558](https://doi.org/https://doi.org/10.1016/j.scitotenv.2023.168558)

3. Xiao, Zufei, Yuan Zhang, Gang Li, Laura J. Carter, Hongtao Wang, Jing Ding, Faith Ka Shun Chan, Yilong Hao, Yaoyang Xu. 2021. “Application of pharmaceutical waste sludge compost alters the antibiotic resistome in soil under the Chinese cabbage system.” *Journal of Cleaner Production* 291: 125229. [https://doi.org/10.1016/j.jclepro.2020.125229](https://doi.org/https://doi.org/10.1016/j.jclepro.2020.125229)

4. Zhu, Dong, Manuel Delgado-Baquerizo, Jian-Qiang Su, Jing Ding, Hu Li, Michael R. Gillings, Josep Penuelas, Yong-Guan Zhu. 2021. “Deciphering potential roles of earthworms in mitigation of antibiotic resistance in the soils from diverse ecosystems.” *Environmental Science & Technology* 55: 7445–7455. [https://doi.org/10.1021/acs.est.1c00811](https://doi.org/https://doi.org/10.1021/acs.est.1c00811)

5. Bai, Defeng, Tong Chen, Jiani Xun, Chuang Ma, Hao Luo, Haifei Yang, Chen Cao, et al. 2025. “EasyMetagenome: A user-friendly and flexible pipeline for shotgun metagenomic analysis in microbiome research.” *iMeta* 4: e70001. [https://doi.org/10.1002/imt2.70001](https://doi.org/https://doi.org/10.1002/imt2.70001)

6. Ewels, Philip, Måns Magnusson, Sverker Lundin, Max Käller. 2016. “MultiQC: summarize analysis results for multiple tools and samples in a single report.” *Bioinformatics* 32: 3047–3048. [https://doi.org/10.1093/bioinformatics/btw354](https://doi.org/https://doi.org/10.1093/bioinformatics/btw354)

7. Blanco-Míguez, Aitor, Francesco Beghini, Fabio Cumbo, Lauren J. McIver, Kelsey N. Thompson, Moreno Zolfo, Paolo Manghi, et al. 2023. “Extending and improving metagenomic taxonomic profiling with uncharacterized species using MetaPhlAn 4.” *Nature Biotechnology* 41: 1633–1644. [https://doi.org/10.1038/s41587-023-01688-w](https://doi.org/https://doi.org/10.1038/s41587-023-01688-w)

8. Beghini, Francesco, Lauren J. McIver, Aitor Blanco-Míguez, Leonard Dubois, Francesco Asnicar, Sagun Maharjan, Ana Mailyan, et al. 2021. “Integrating taxonomic, functional, and strain-level profiling of diverse microbial communities with bioBakery 3.” *eLife* 10: e65088. [https://doi.org/10.7554/eLife.65088](https://doi.org/https://doi.org/10.7554/eLife.65088)

9. Yin, Xiaole, Xiawan Zheng, Liguan Li, An-Ni Zhang, Xiao-Tao Jiang, Tong Zhang. 2023. “ARGs-OAP v3.0: antibiotic-resistance gene database curation and analysis pipeline optimization.” *Engineering* 27: 234–241. [https://doi.org/10.1016/j.eng.2022.10.011](https://doi.org/https://doi.org/10.1016/j.eng.2022.10.011)

10. Yin, Xiaole, Xi Chen, Xiao-Tao Jiang, Ying Yang, Bing Li, Marcus Ho-Hin Shum, Tommy T. Y. Lam, et al. 2023. “Toward a universal unit for quantification of antibiotic resistance genes in environmental samples.” *Environmental Science & Technology* 57: 9713–9721. [https://doi.org/10.1021/acs.est.3c00159](https://doi.org/https://doi.org/10.1021/acs.est.3c00159)

11. Bertrand, Denis, Jim Shaw, Manesh Kalathiyappan, Amanda Hui Qi Ng, M. Senthil Kumar, Chenhao Li, Mirta Dvornicic, et al. 2019. “Hybrid metagenomic assembly enables high-resolution analysis of resistance determinants and mobile elements in human microbiomes.” *Nature Biotechnology* 37: 937–944. [https://doi.org/10.1038/s41587-019-0191-2](https://doi.org/https://doi.org/10.1038/s41587-019-0191-2)

12. Uritskiy, Gherman V., Jocelyne DiRuggiero, James Taylor. 2018. “MetaWRAP—a flexible pipeline for genome-resolved metagenomic data analysis.” *Microbiome* 6: 158. [https://doi.org/10.1186/s40168-018-0541-1](https://doi.org/https://doi.org/10.1186/s40168-018-0541-1)

13. Chklovski, Alex, Donovan H. Parks, Ben J. Woodcroft, Gene W. Tyson. 2023. “CheckM2: a rapid, scalable and accurate tool for assessing microbial genome quality using machine learning.” *Nature Methods* 20: 1203–1212. [https://doi.org/10.1038/s41592-023-01940-w](https://doi.org/https://doi.org/10.1038/s41592-023-01940-w)

14. Alcock, Brian P, William Huynh, Romeo Chalil, Keaton W Smith, Amogelang R Raphenya, Mateusz A Wlodarski, Arman Edalatmand, et al. 2023. “CARD 2023: expanded curation, support for machine learning, and resistome prediction at the comprehensive antibiotic resistance database.” *Nucleic Acids Research* 51(D1): D690–D699. [https://doi.org/ 10.1093/nar/gkac920](https://doi.org/https://doi.org/10.1093/nar/gkz935)

15. Arango-Argoty, Gustavo, Emily Garner, Amy Pruden, Lenwood S. Heath, Peter Vikesland, Liqing Zhang. 2018. “DeepARG: a deep learning approach for predicting antibiotic resistance genes from metagenomic data.” *Microbiome* 6: 23. [https://doi.org/10.1186/s40168-018-0401-z](https://doi.org/https://doi.org/10.1186/s40168-018-0401-z)

16. Brown, Connor L., James Mullet, Fadi Hindi, James E. Stoll, Suraj Gupta, Minyoung Choi, Ishi Keenum, Peter Vikesland, Amy Pruden, Liqing Zhang. 2022. “mobileOG-db: a manually curated database of protein Families mediating the life cycle of bacterial mobile genetic elements.” *Applied and Environmental Microbiology* 88: e00991–00922. [https://doi.org/10.1128/aem.00991-22](https://doi.org/https://doi.org/10.1128/aem.00991-22)

17. Steinegger, Martin, Johannes Söding. 2017. “MMseqs2 enables sensitive protein sequence searching for the analysis of massive data sets.” *Nature Biotechnology* 35: 1026–1028. [https://doi.org/10.1038/nbt.3988](https://doi.org/https://doi.org/10.1038/nbt.3988)

18. Zheng, Dongsheng, Guoyu Yin, Min Liu, Lijun Hou, Yi Yang, Thomas P. Van Boeckel, Yanling Zheng, Ye Li. 2022. “Global biogeography and projection of soil antibiotic resistance genes.” *Science Advances* 8: eabq8015. [https://doi.org/10.1126/sciadv.abq8015](https://doi.org/https://doi.org/10.1126/sciadv.abq8015)

19. Letunic, Ivica, Peer Bork. 2024. “Interactive Tree of Life (iTOL) v6: recent updates to the phylogenetic tree display and annotation tool.” *Nucleic Acids Research* 52: W78–W82. [https://doi.org/10.1093/nar/gkae268](https://doi.org/https://doi.org/10.1093/nar/gkae268)

20. de Vries, Franciska T., Rob I. Griffiths, Mark Bailey, Hayley Craig, Mariangela Girlanda, Hyun Soon Gweon, Sara Hallin, et al. 2018. “Soil bacterial networks are less stable under drought than fungal networks.” *Nature Communications* 9: 3033. [https://doi.org/10.1038/s41467-018-05516-7](https://doi.org/https://doi.org/10.1038/s41467-018-05516-7)

21. Chen, Tong, Yong-Xin Liu, Luqi Huang. 2022. “ImageGP: An easy-to-use data visualization web server for scientific researchers.” *iMeta* 1: e5. [https://doi.org/10.1002/imt2.5](https://doi.org/https://doi.org/10.1002/imt2.5)

22. Jiao, Shuo, Weimin Chen, Jieli Wang, Nini Du, Qiaoping Li, Gehong Wei. 2018. “Soil microbiomes with distinct assemblies through vertical soil profiles drive the cycling of multiple nutrients in reforested ecosystems.” *Microbiome* 6: 146. [https://doi.org/10.1186/s40168-018-0526-0](https://doi.org/https://doi.org/10.1186/s40168-018-0526-0)

**
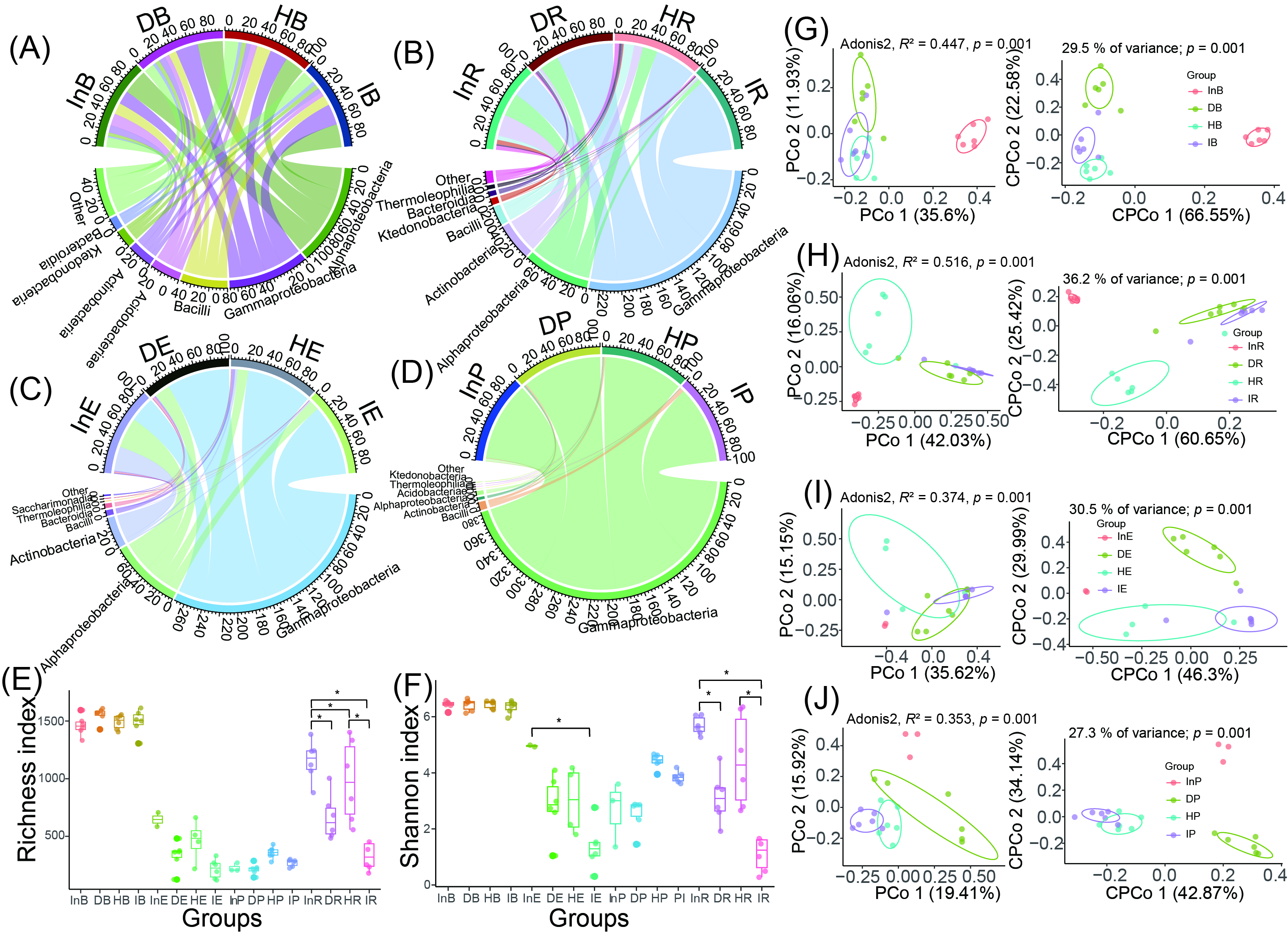
**

Figure S1 Response of bacterial communities to *R. solanacearum* infestation in the soil–tomato continuum. Distribution patterns and class-level compositions of bacterial communities are shown for (A and G) bulk soil, (B and H) rhizosphere, (C and I) root endosphere, and (D and J) phyllosphere across four plant health states: before onset, healthy, infected, and dead. Panels (E) and (F) display bacterial ZOTU richness and Shannon diversity, respectively, across the same four states. Abbreviations: HB, HR, HE, HP = bulk soil, rhizosphere, root endosphere, and phyllosphere of healthy plants; IB, IR, IE, IP = corresponding niches of infected plants; DB, DR, DE, DP = niches of dead plants; InB, InR, InE, InP = niches before onset. Statistical significance was determined using one-way ANOVA followed by Tukey’s HSD post hoc test. Adjusted *p*-values: *, < 0.05; **, < 0.01


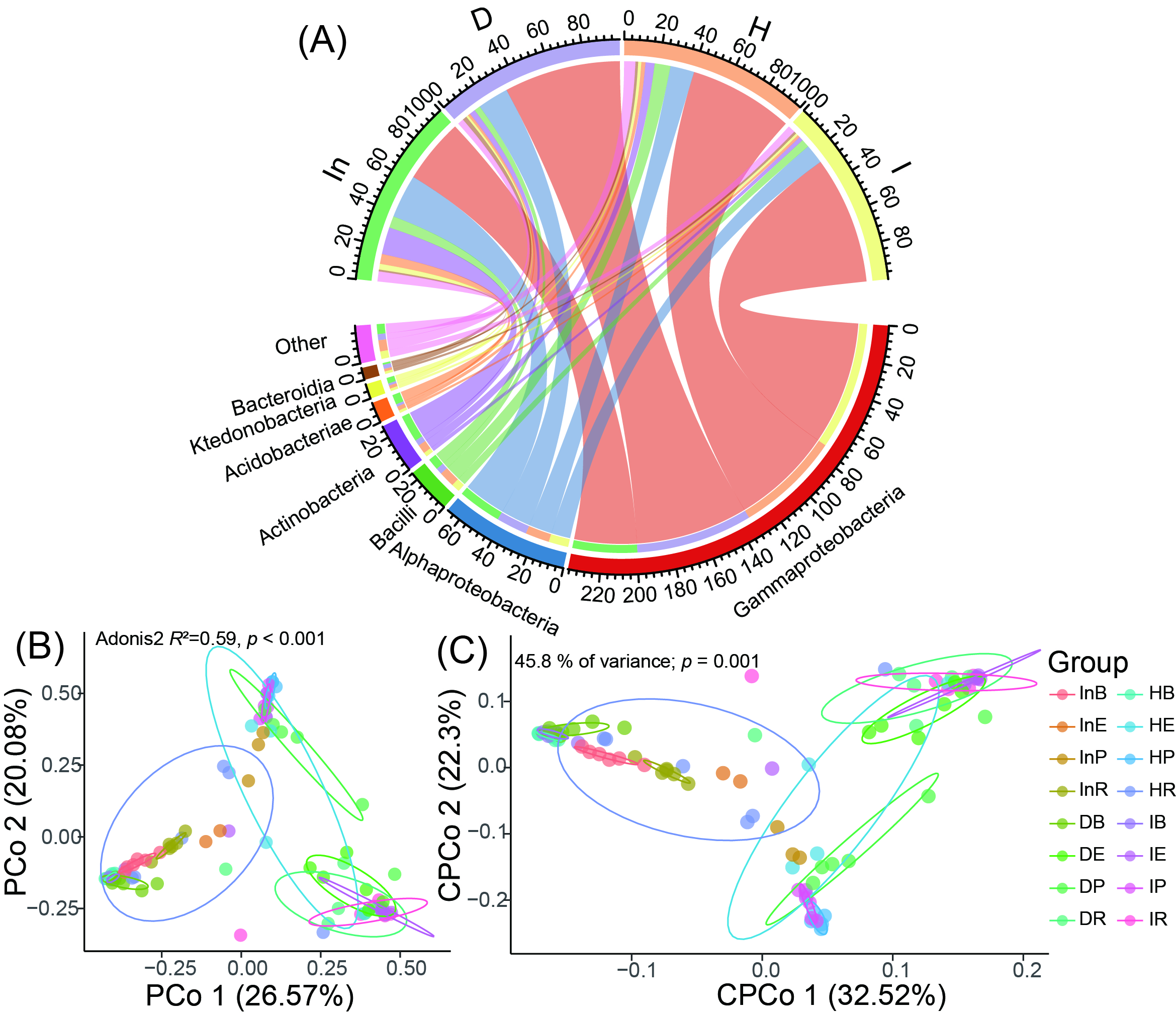


Figure S2 Bacterial community responses to *R. solanacearum* infestation in the soil–tomato continuum. (A) Chord diagram illustrating the distribution of bacterial classes across soil–tomato compartments under different physiological states. (B) PCoA and (C) CPCoA depicting β-diversity patterns of bacterial communities across the soil–tomato continuum.


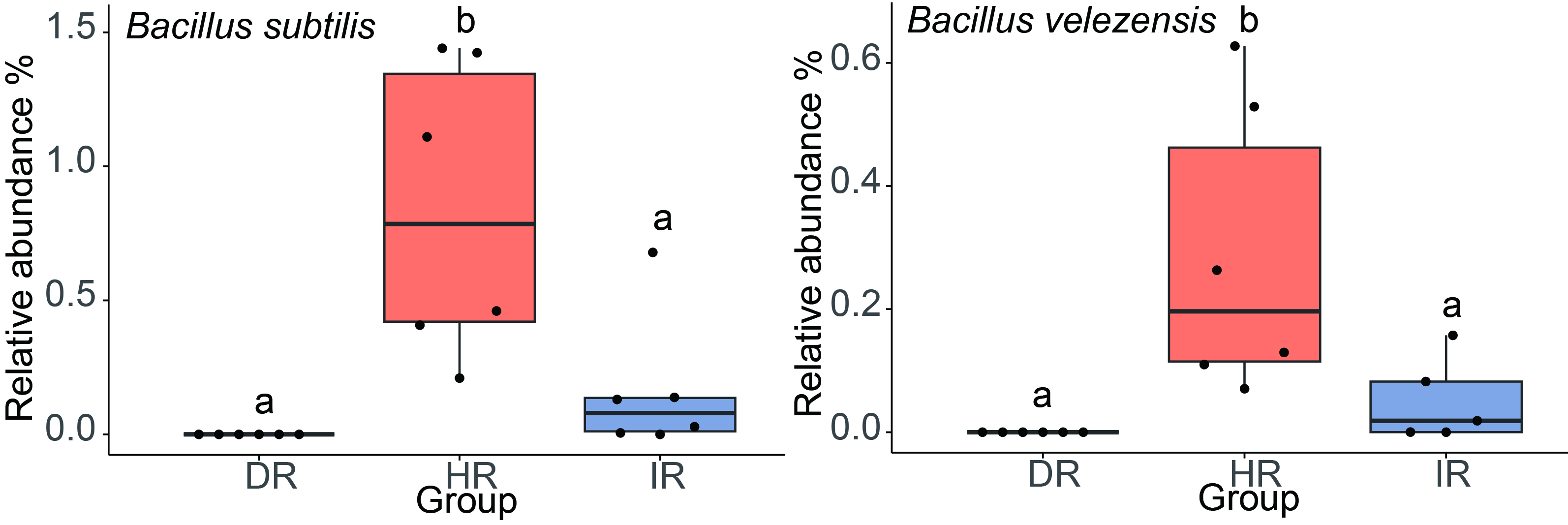


Figure S3 Relative abundance of *Bacillus subtilis* and *Bacillus velezensis* in rhizosphere soils from healthy, infected, and dead tomato plants. Box plots illustrate significant differences among plant health states, determined using one-way ANOVA followed by Tukey’s HSD post hoc test (*p* < 0.05).


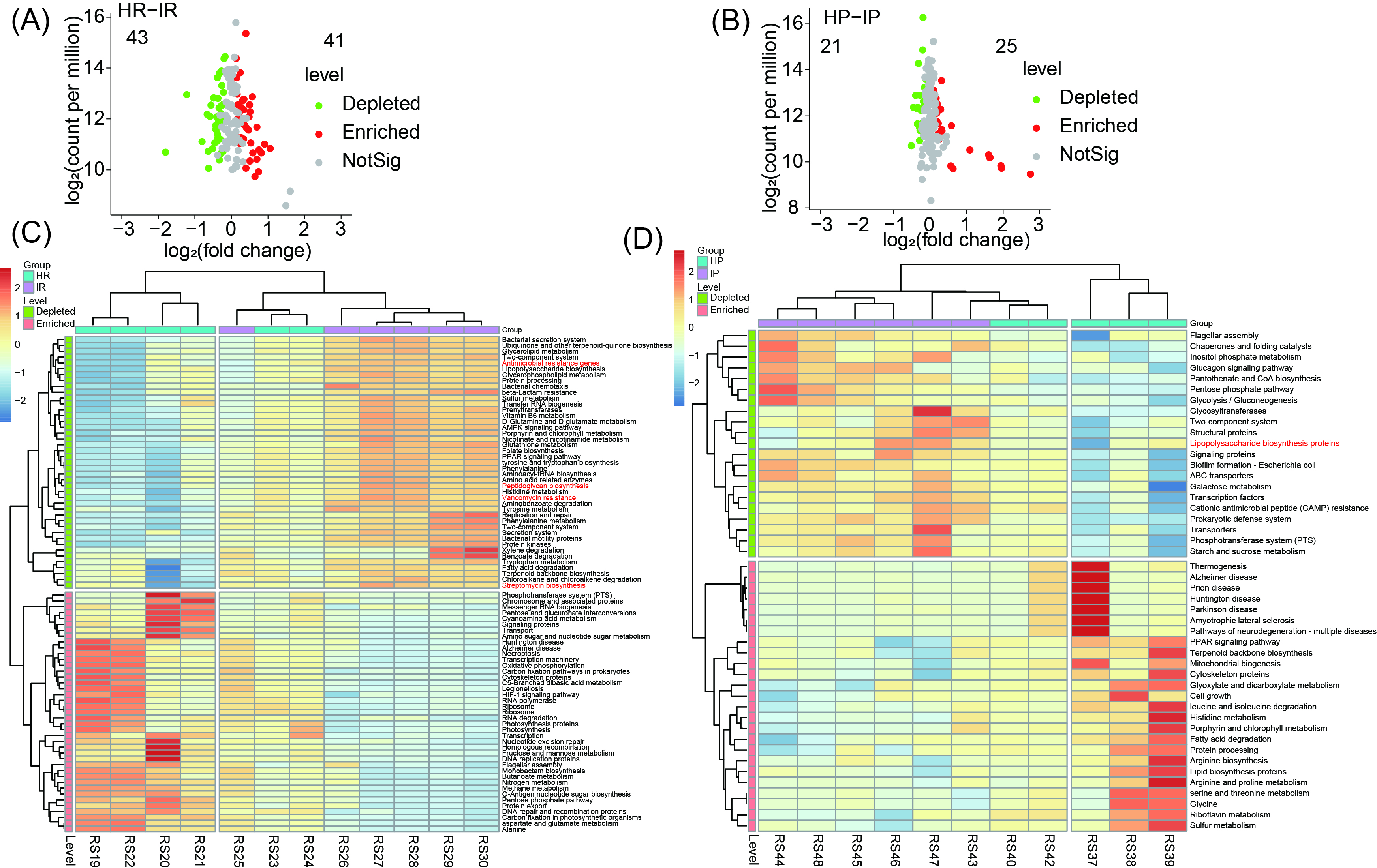


Figure S4 Differential metabolic pathways between healthy and infected plants in the soil–tomato continuum. Volcano plots display significantly enriched pathways in the rhizosphere (A) and phyllosphere (B). Heatmaps show corresponding Log₂FC of enriched pathways in the rhizosphere (C) and phyllosphere (D).


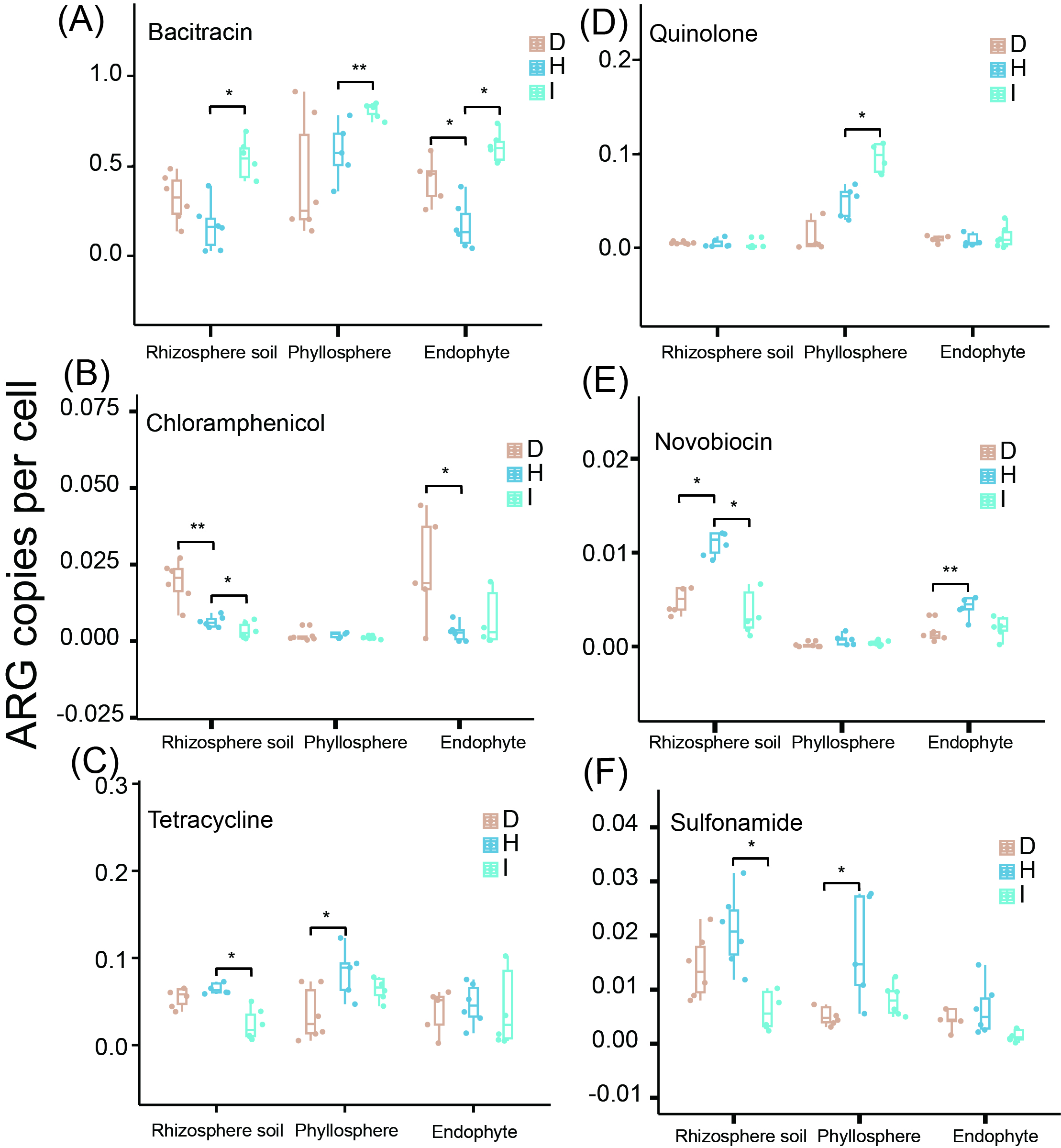


Figure S5 Relative abundance of ARG types across plant health states based on metagenomic analysis. Box plots illustrate variations among healthy (H), infected (I), and dead (D) plants. Statistical significance was determined using one-way ANOVA followed by Tukey’s HSD post hoc test. Adjusted *p*-values: *, < 0.05; **, < 0.01.


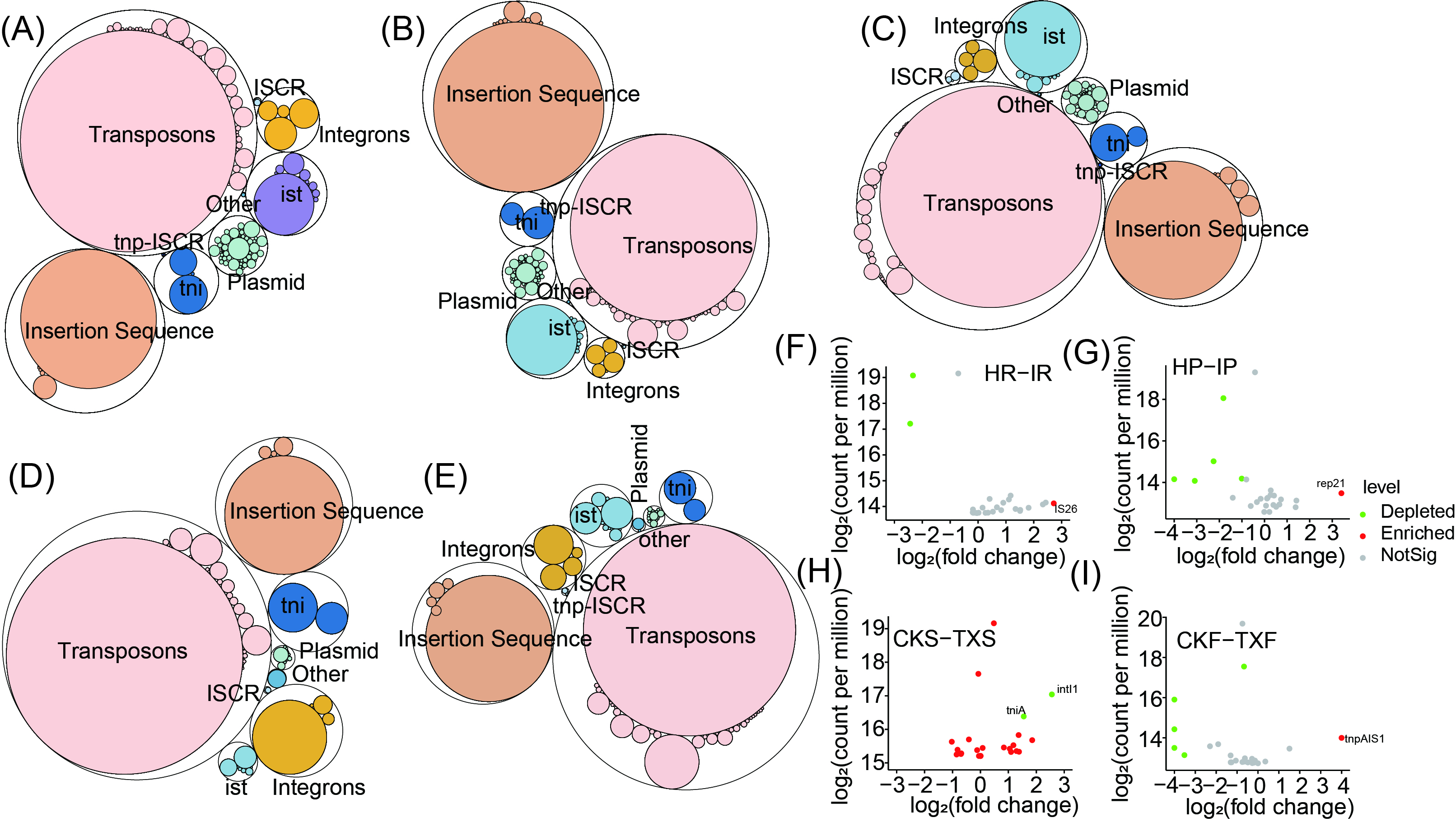


Figure S6 Distribution and composition of MGEs in the soil–tomato and soil–strawberry continua. MGE compositions are color-coded by type, with inner and outer circles representing subtypes and types, respectively. Circle sizes indicate normalized abundance for: (A) healthy, (B) infected, (C) dead, (D) CK, and (E) TX groups. Volcano plots illustrate the impact of *R. solanacearum* infestation on MGEs in the soil–tomato continuum (F, rhizosphere soil and G, phyllosphere) and changes in the soil–strawberry continuum resulting from the soil-borne legacy of bacterial wilt (*n* = 6 per group): (H) rhizosphere soil and (I) strawberry fruit.


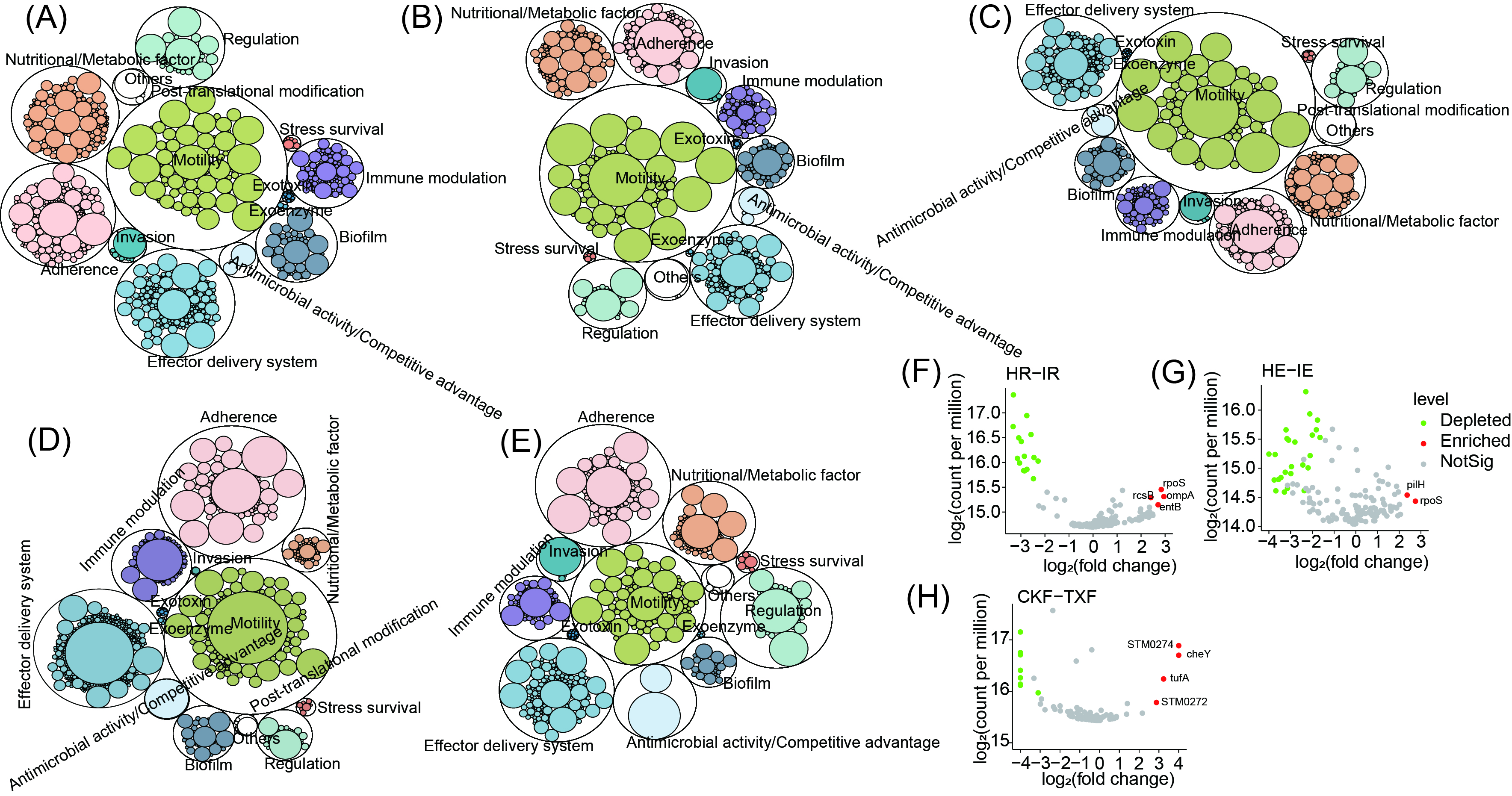


Figure S7 Distribution and composition of VFGs in the soil–tomato and soil–strawberry continua. VFG compositions are color-coded by type, with inner and outer circles representing subtypes and types, respectively. Circle sizes indicate normalized abundance for: (A) healthy, (B) infected, (C) dead, (D) CK, and (E) TX groups. Volcano plots illustrate the impact of *R. solanacearum* infestation on VFGs in the soil–tomato continuum (F, rhizosphere soil and G, root endosphere) and changes in the soil–strawberry continuum resulting from the soil-borne legacy of bacterial wilt (*n* = 6 per group), with (H) representing strawberry fruits.


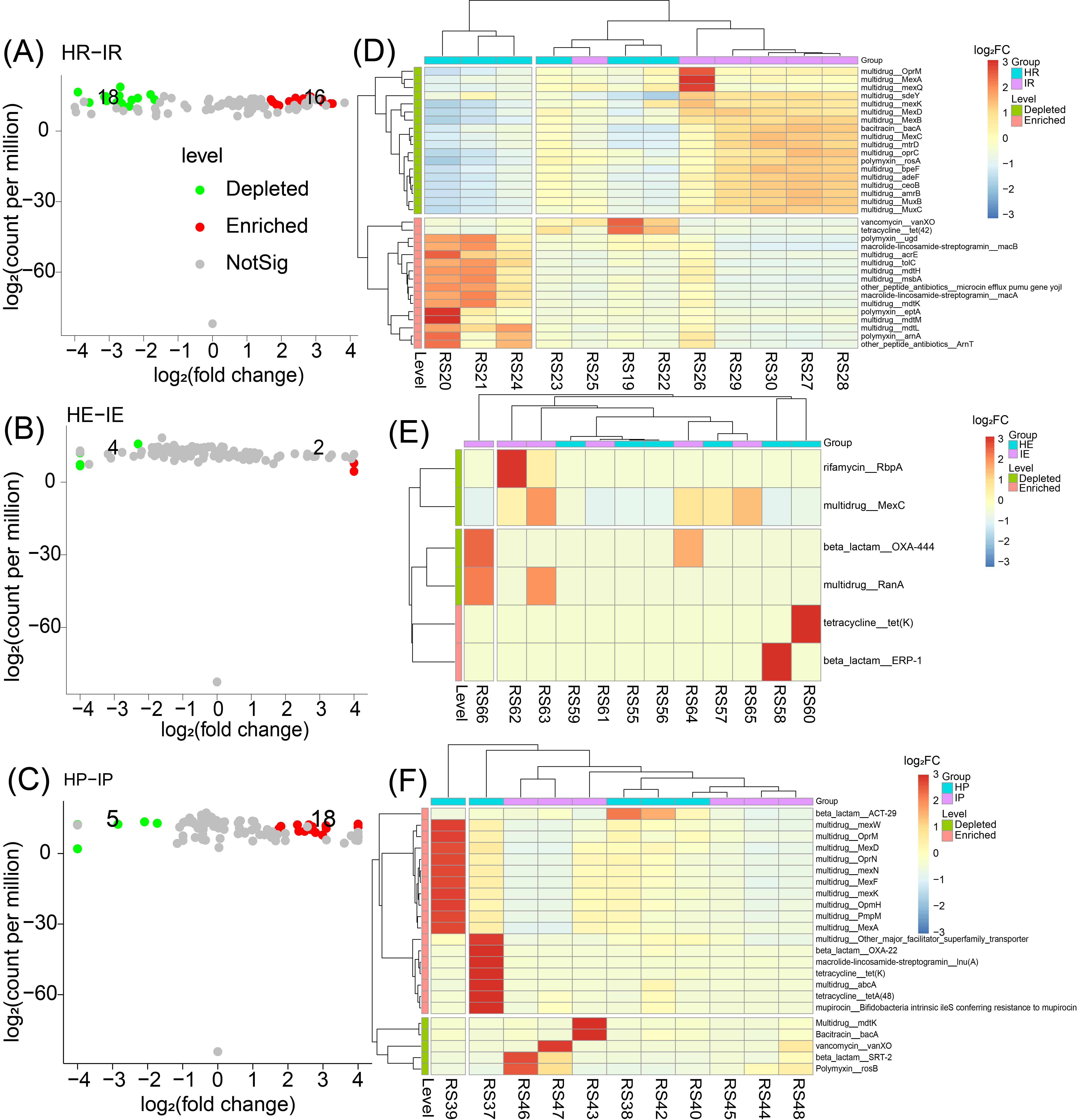


Figure S8 Differential ARG types between healthy and infected plants in the soil–tomato continuum. Volcano plots depict significantly enriched ARG types in the rhizosphere (A), root endophytes (B), and phyllosphere (C). Corresponding heatmaps show Log₂FC of enriched ARG subtypes in the rhizosphere (D), root endophytes (E), and phyllosphere (F).


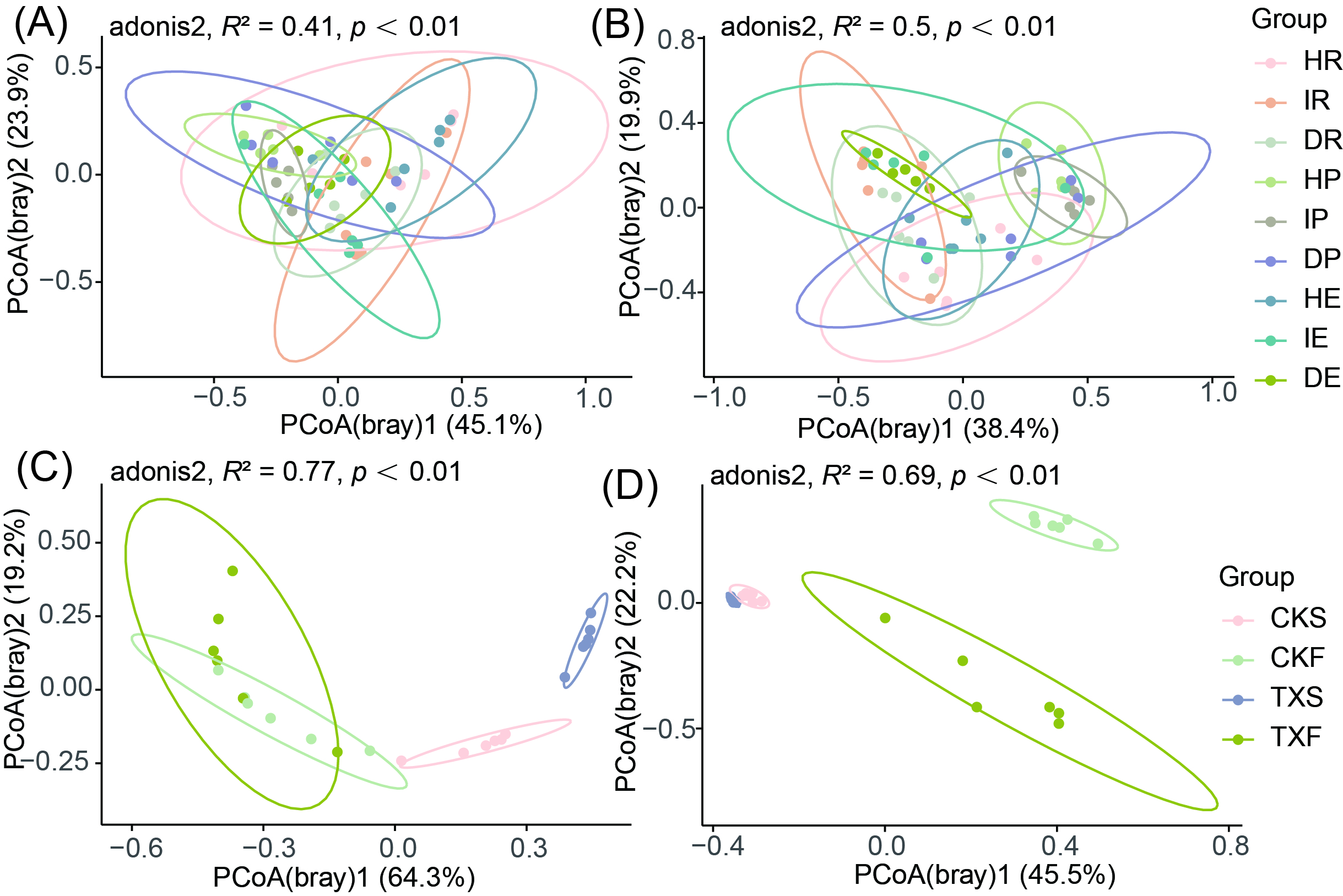


Figure S9 Response of MGEs and VFGs to *R. solanacearum* infestation. PCoA depicts the distribution patterns of MGEs (A) and VFGs (B) in the soil–tomato continuum under different plant health states, and MGEs (C) and VFGs (D) in the soil–strawberry continuum influenced by the soil-borne legacy of bacterial wilt.


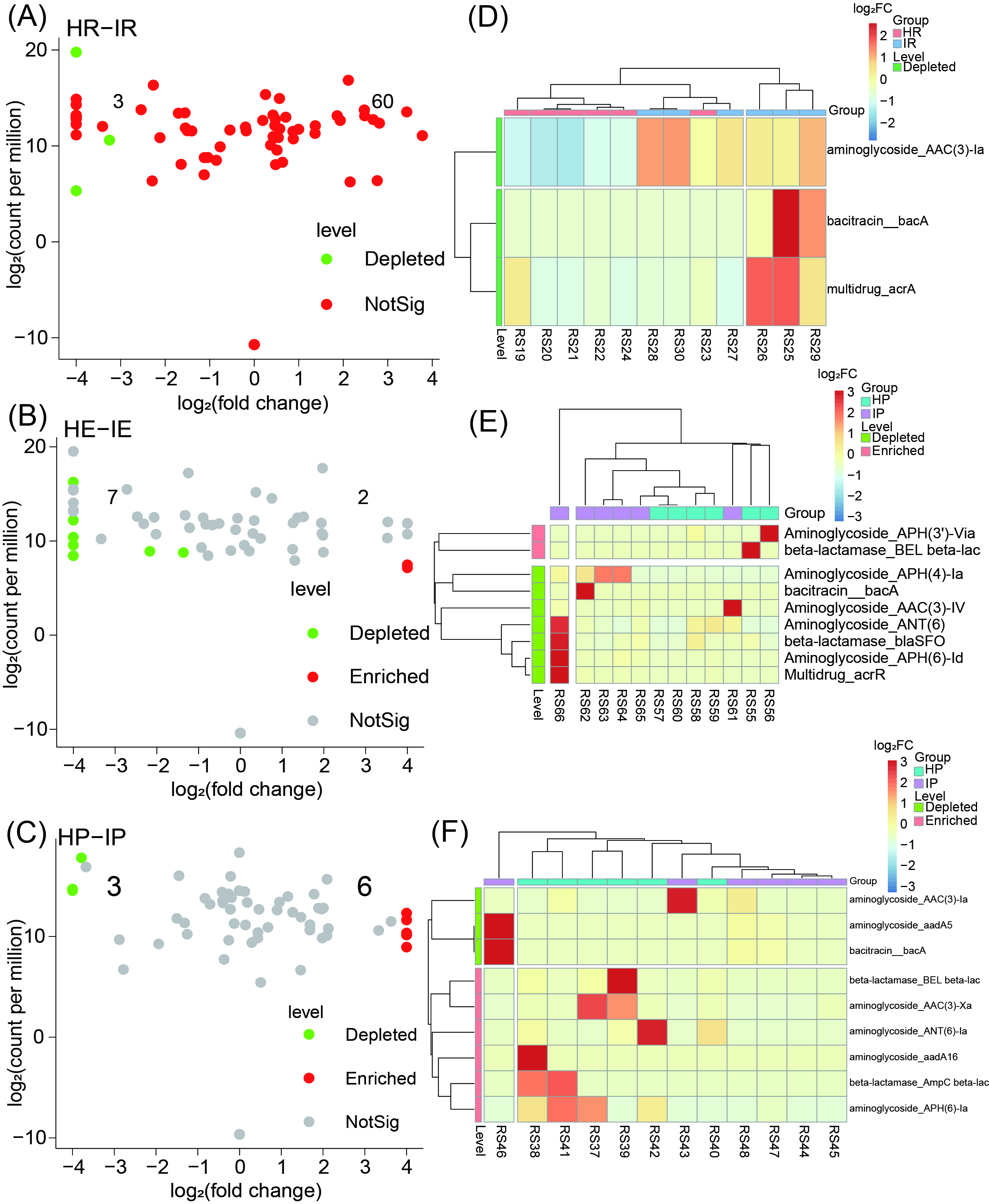


Figure S10 Differential ARG profiles between healthy and infected plants based on HT-qPCR. Volcano plots illustrate significantly different ARG types in the soil–tomato continuum: (A) rhizosphere, (B) root endophyte, and (C) phyllosphere. Heatmaps display Log₂FC of enriched ARG subtypes in corresponding compartments: (D) rhizosphere, (E) root endophyte, and (F) phyllosphere.


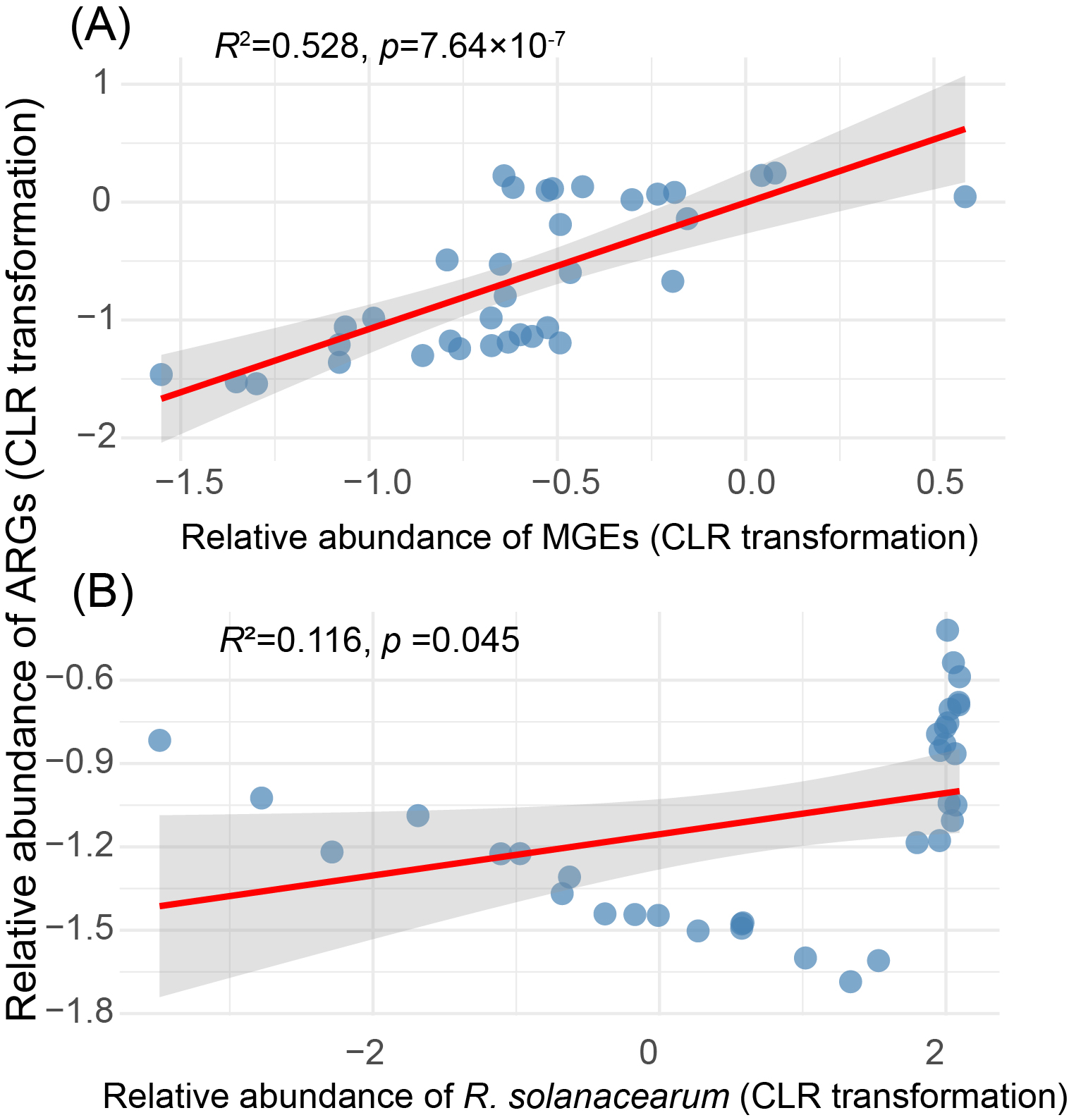


Figure S11 Linear regression analysis linking ARGs with MGEs and *R. solanacearum*. Relationships between ARG relative abundance and that of (A) MGEs and (B) *R. solanacearum*.


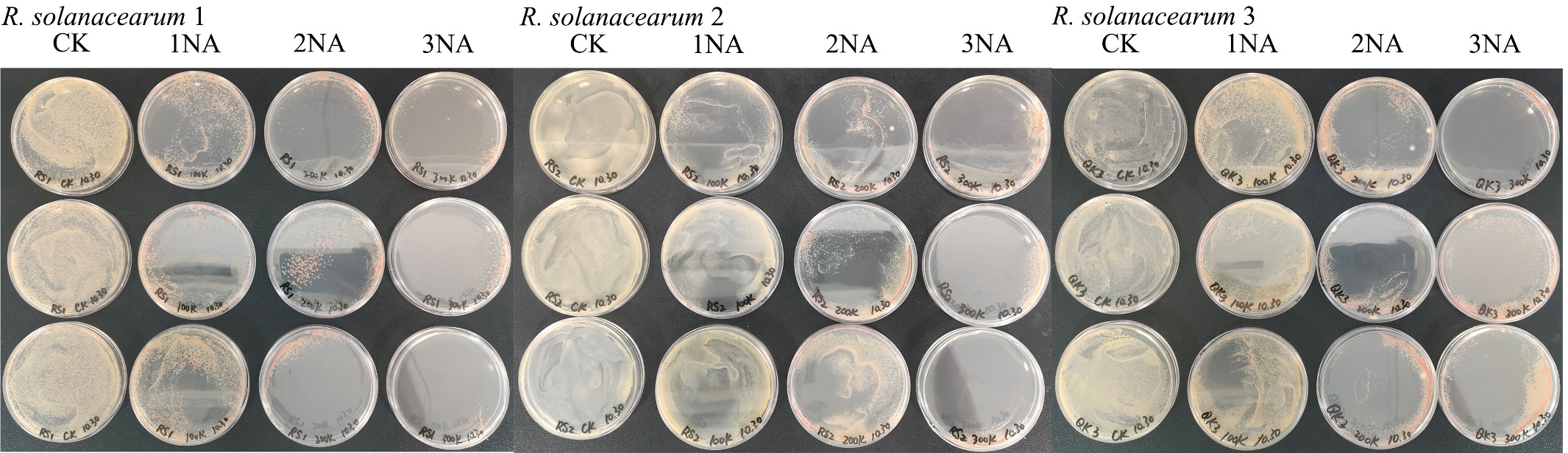


Figure S12 Antibiotic susceptibility test results for *R. solanacearum*.


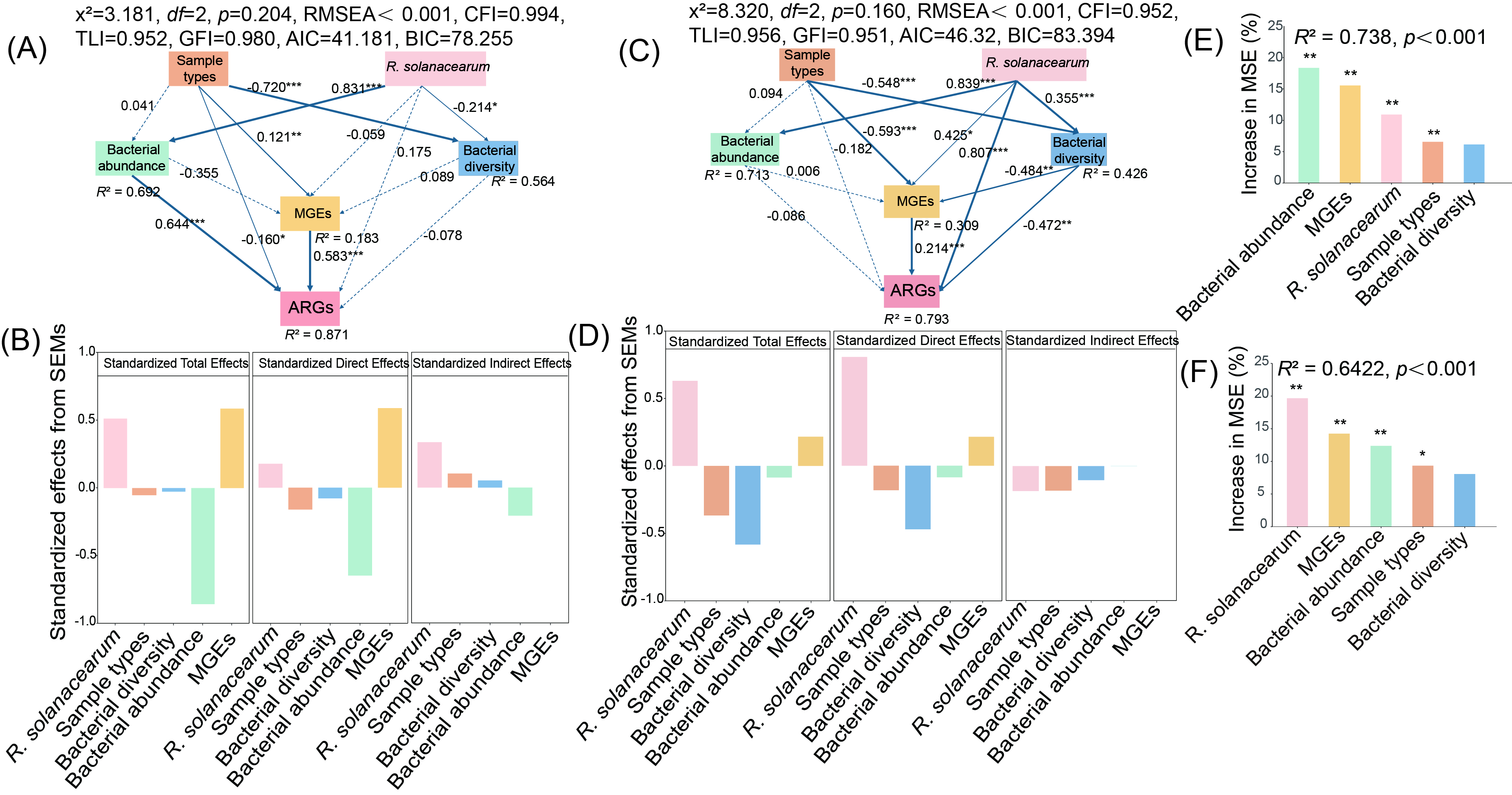


Figure S13 Potential drivers of ARG variation in the soil–plant continuum identified by metagenomic and HT-qPCR analyses. SEM illustrates the direct and indirect effects of key ecological and microbial factors on ARG abundance, with standardized total, direct, and indirect effects shown for (A and B) metagenomic data and (C and D) HT-qPCR data. RF analysis ranks the relative importance of predictors of ARGs based on the percentage increase in mean square error for (E) metagenomic and (F) HT-qPCR datasets.


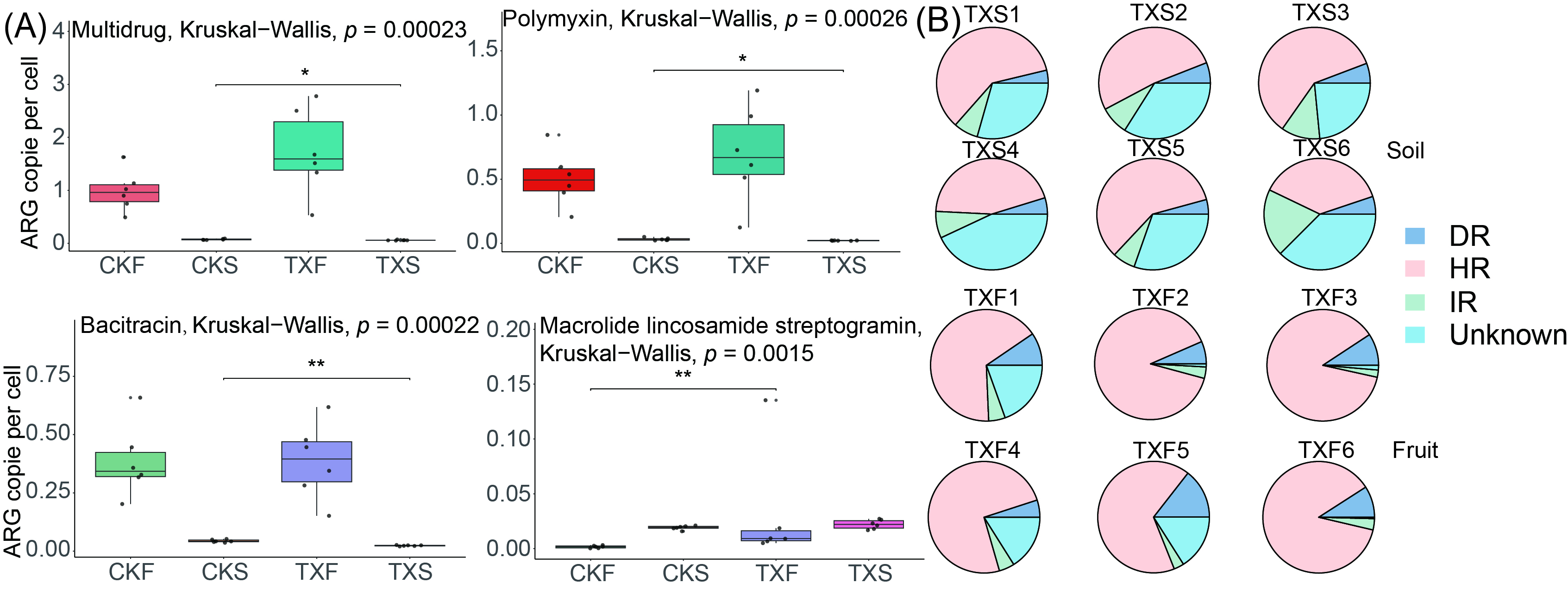


Figure S14 Distribution and niche-specific patterns of ARGs revealed by metagenomic analysis. (A) Boxplots showing the relative abundance of multidrug, polymyxin, bacitracin, and MLS resistance genes across different ecological niches. (B) Venn diagram illustrating the overlap and unique distribution of ARGs among three sample groups (IR + DR, HR, and TXR + TXF), with each circle representing one group.

**
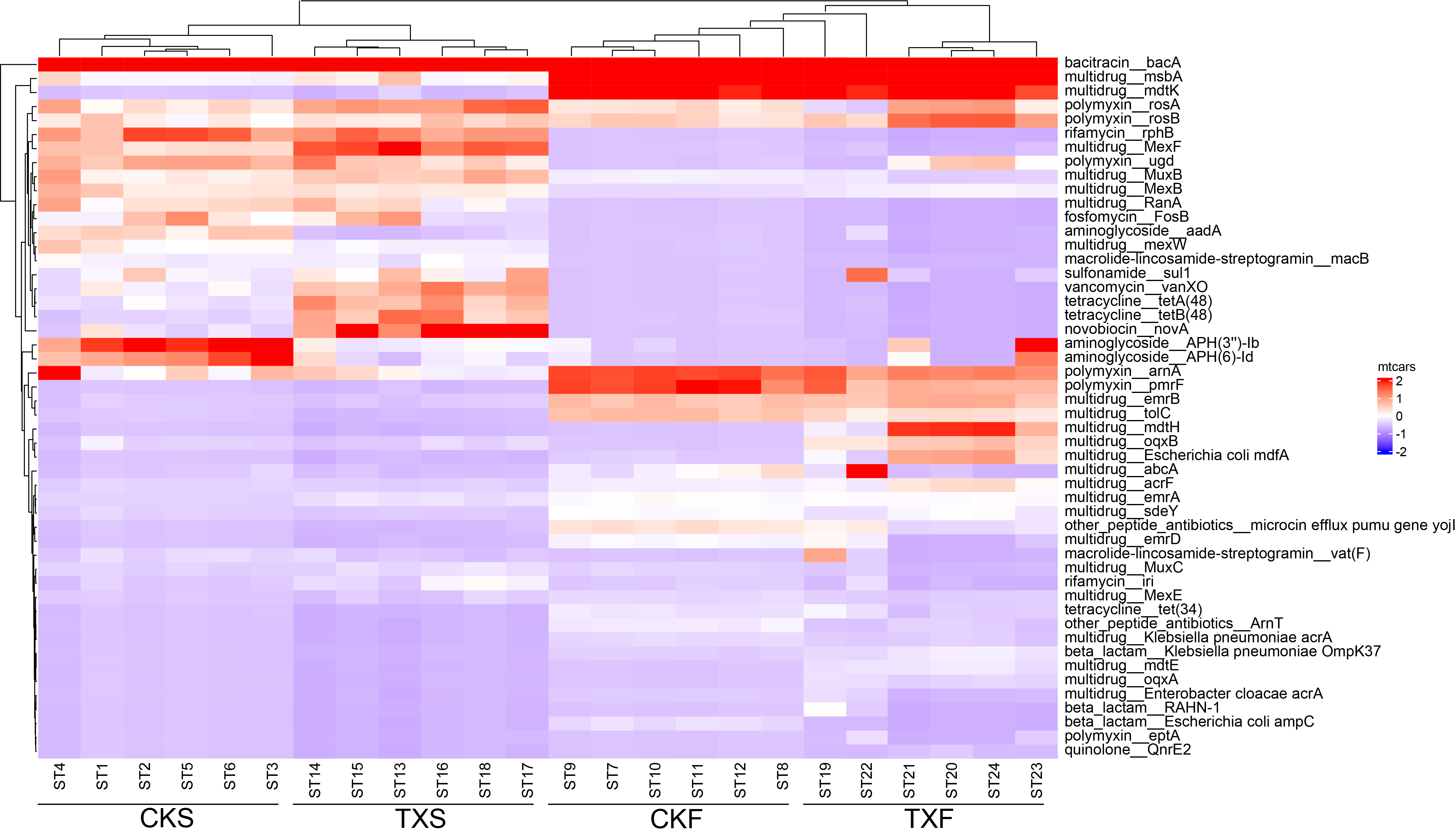
**

Figure S15 Heatmap illustrating the top 50 most abundant ARG subtypes in the soil–strawberry continuum. ARG subtypes are ranked by their mean relative abundance across all sample types.


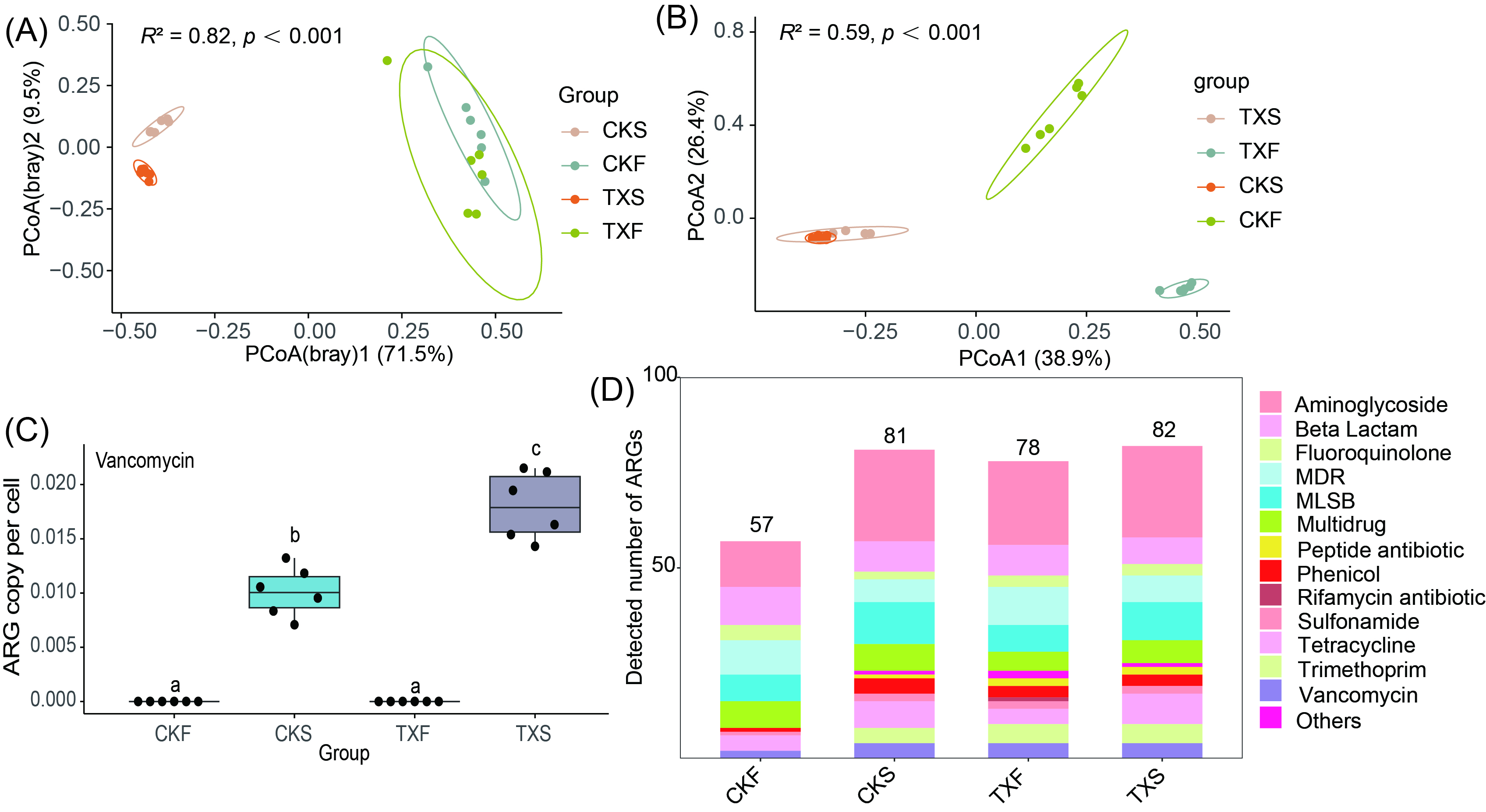


Figure S16 Distribution and composition of ARGs in the soil–strawberry continuum. (A and B) PCoA illustrating the distribution patterns of ARGs associated with the soil-borne legacy of bacterial wilt, based on metagenomic and HT-qPCR datasets, respectively. (C) Boxplots showing the relative abundance of vancomycin resistance genes. (D) Boxplots presenting the total number of ARGs detected in the soil–strawberry continuum with a bacterial wilt legacy.


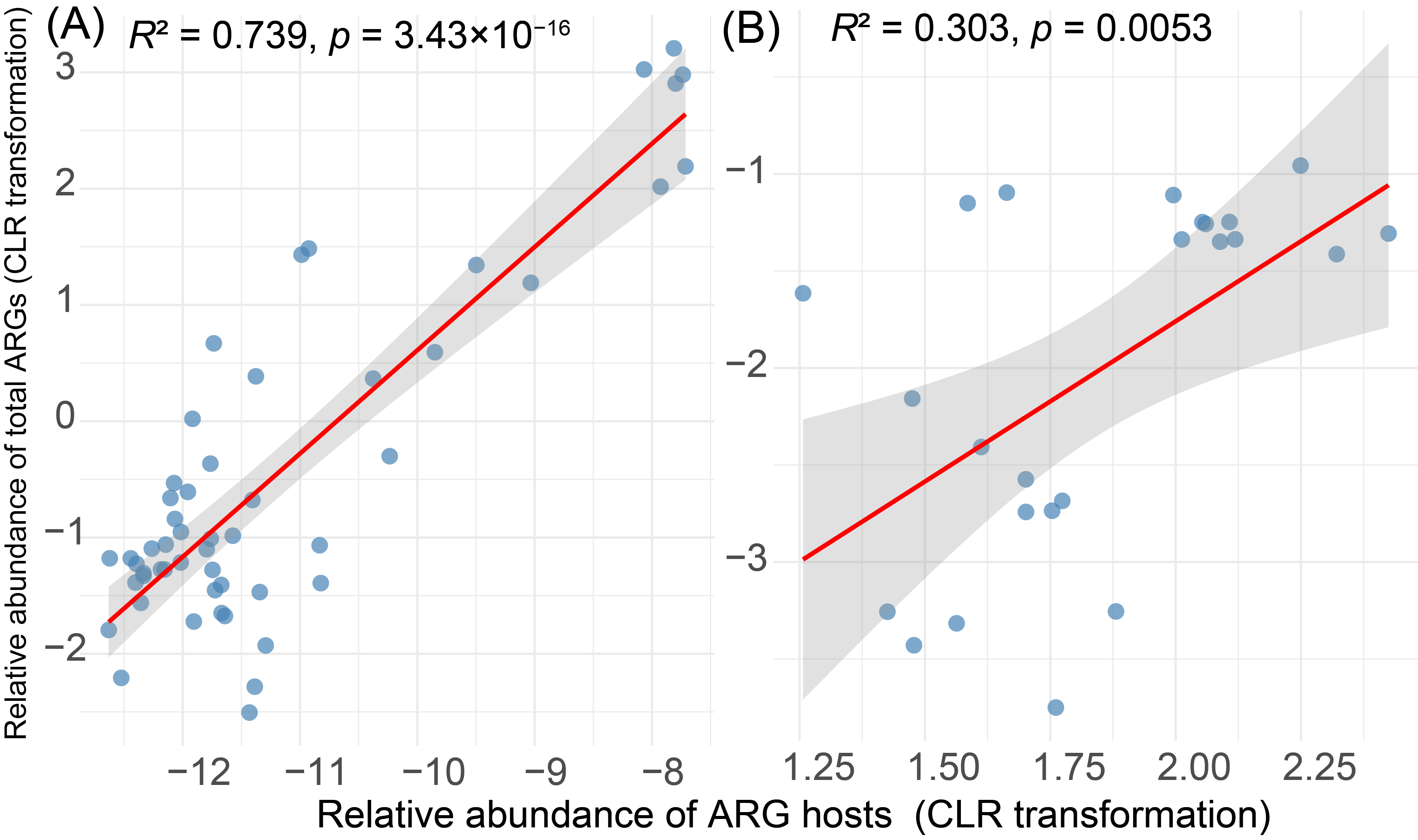


Figure S17 Linear regression analysis of the relationship between ARG relative abundance and ARG-host relative abundance in the soil–tomato (A) and soil–strawberry (B) continua.


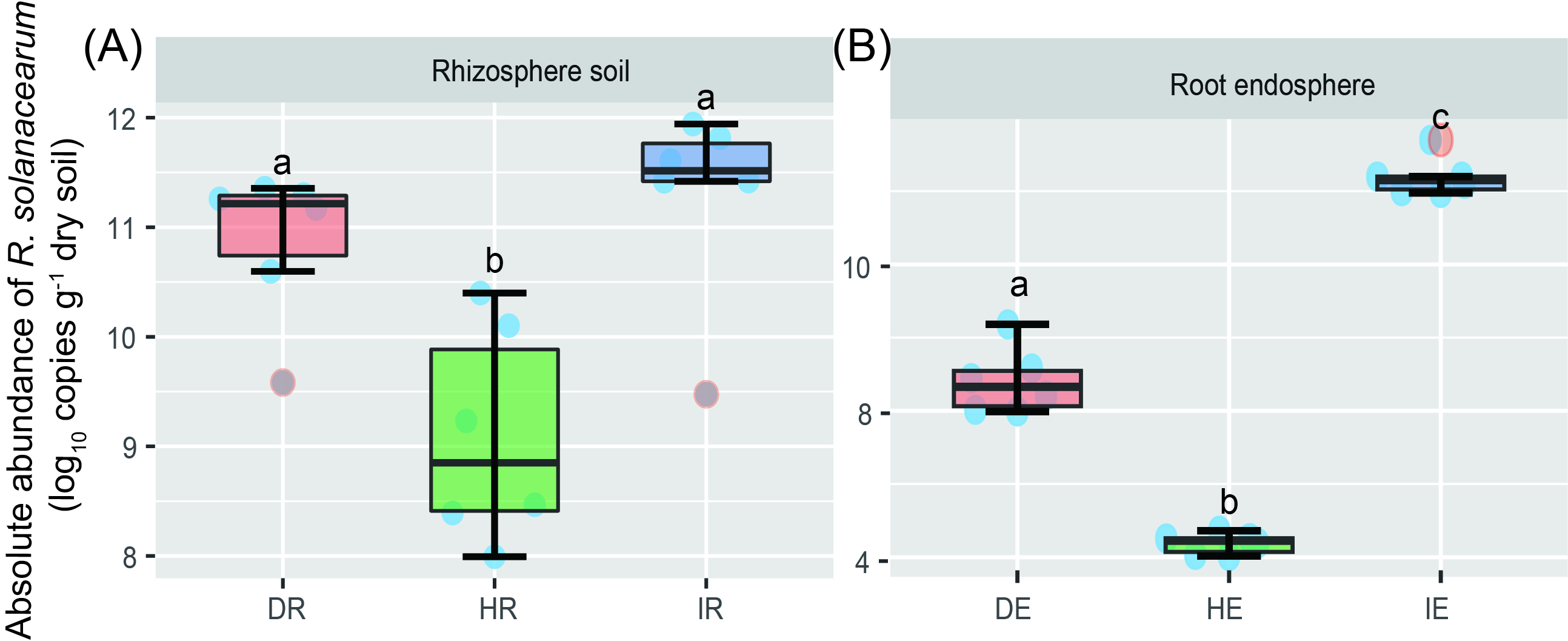


Figure S18 Absolute abundance of *R. solanacearum* in rhizosphere soil (A) and root endophytes (B) of healthy, infected, and dead tomato plants.


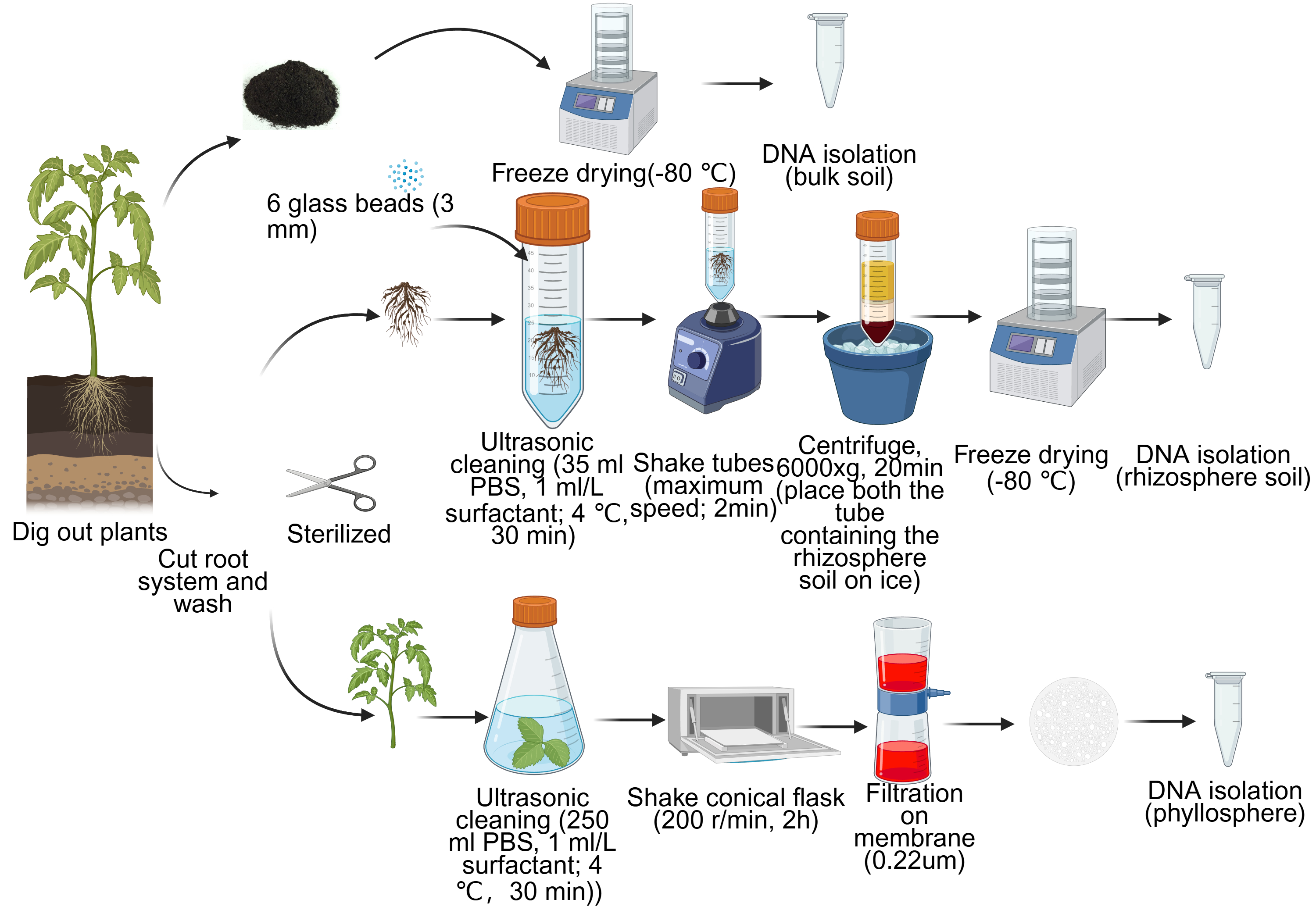


Figure S19 Schematic of fractionation protocol.
